# Trauma and anxiety interactions relate to reward processing in adolescents

**DOI:** 10.1016/j.jad.2025.03.076

**Published:** 2025-03-14

**Authors:** Alexa N. Duran, Ruiyu Yang, Madelin Gredvig, Jillian Lee Wiggins

**Affiliations:** a Department of Psychology, San Diego State University, San Diego, CA 92182, USA; b San Diego State University, University of California San Diego Joint Doctoral Program in Clinical Psychology, USA

**Keywords:** Adolescent, Anxiety, Risk, Trauma exposure, Vulnerability, Reward processing

## Abstract

Anxiety is highly prevalent among adolescents, often linked to trauma exposure. However, not all youth with trauma develop anxiety, suggesting variability in risk pathways. This study investigates how neural reward system alterations may signal vulnerability to anxiety following trauma. We hypothesized that differences in reward-related connectivity in the ventral striatum and amygdala would distinguish adolescents with higher trauma and higher anxiety (anxiety risk group) from those with higher trauma but lower anxiety (non-anxiety development), as well as from youth with lower trauma and lower anxiety (typical development) and with lower trauma and higher anxiety (non-trauma-related anxiety). We utilized a sample of 44 adolescents (ages 11–19) with varying levels of trauma exposure and anxiety who completed a child-friendly monetary incentive delay task and analyzed their amygdala and ventral striatum functional connectivity during the task to assess the interaction between trauma exposure and anxiety levels. Our findings reveal distinct Trauma × Anxiety neural connectivity patterns in widespread prefrontal, temporal, and occipital clusters, potentially serving as biomarkers for anxiety risk. These results provide insight into potential neurobiological markers associated with anxiety vulnerability, bringing us a step closer to identifying targets for future intervention development. By highlighting unique patterns of reward processing associated with different trauma and anxiety profiles, this study advances our knowledge of the neural mechanisms underlying anxiety risk, laying the groundwork for future research on neurobiologically informed approaches to prevention and treatment.

## 1. Introduction

Anxiety symptoms are among the most prevalent and debilitating complaints in pediatric psychiatry, with lifetime disorder rates as high as 1 in 3 by adolescence (Merikangas et al., 2010). Childhood trauma is one of the most potent risk factors for developing anxiety (Clark et al., 2010; Cohen et al., 2001; Green et al., 2010; Kessler et al., 2010; Li et al., 2016), yet the relationship between trauma and anxiety is complex, as not all adolescents with trauma develop anxiety, and anxiety can arise from multiple pathways beyond trauma exposure. Identifying neurobiological mechanisms that contribute to these different trajectories could enhance our understanding of anxiety risk. While PTSD and depression are common consequences of trauma, this study focuses on anxiety due to its high prevalence and transdiagnostic significance (Kessler et al., 2010). Although PTSD and anxiety share some overlapping symptoms, trauma-related psychopathology is heterogeneous, and brain imaging may offer a window into disentangling the mechanisms underlying these varying trauma and anxiety pathways. Indeed, reward-related neural alterations have been linked with both childhood trauma and anxiety. Here, we leverage a risk-enriched diverse sample to begin to characterize reward-related neural mechanisms that may differentiate varying levels of anxiety and trauma, and by doing so, take a step toward teasing apart heterogeneous pathways of anxiety risk.

Adolescence is a critical window for the onset of anxiety disorders, as the brain undergoes significant maturation, particularly in regions associated with emotion regulation and reward processing (Casey et al., 2019; Pine et al., 2009). Additionally, anxiety disorders are the most common psychiatric conditions during this developmental period (Merikangas et al., 2010). There is evidence that neural activation in regions including ventral striatum, ventromedial prefrontal cortex, and rostral anterior cingulate cortex varies by anxiety levels (Hu, 2018) and adolescents with generalized anxiety disorder exhibit increased functional connectivity between the striatum and amygdala (Anderson et al., 2023), as well as changes in reward-related circuits like the ventral striatum-amygdala (Xie et al., 2021). However, research has been less consistent in demonstrating clear variations in neural associations based on differing anxiety levels. Adolescents with higher anxiety were shown to exhibit reduced ventral striatum activation during reward processing, and some studies have reported diminished responsiveness to monetary rewards in regions such as the caudate and putamen (Kessel et al., 2015; Lahat et al., 2016; Sequeira et al., 2022), suggesting that disrupted functioning in these brain regions may underlie the reduced capacity for reward learning seen in anxious youth.

Childhood trauma is highly prevalent, as 2 in 3 youths experience at least one direct or witnessed victimization (Finkelhor et al., 2009; McLaughlin et al., 2012). Childhood trauma is linked to the onset of various psychopathologies, (Chapman et al., 2004; Clark et al., 2010; Cohen et al., 2001; Green et al., 2010; Kessler et al., 2010; Li et al., 2016; Nanni et al., 2012), and importantly, prior trauma exposure serves as a major risk indicator for the development of anxiety disorders in adolescence (Chiu et al., 2024). Functional neuroimaging studies reveal that maltreated individuals exhibit altered activation in striatal and prefrontal regions during reward anticipation and receipt, and diminished resting-state functional connectivity between the amygdala and cortical regions including anterior cingulate cortex (Teicher et al., 2016; Yang et al., 2021). Additionally key components of the reward circuit, including the mesolimbic and striatal regions with the anterior cingulate cortex, show structural and functional abnormalities. Trauma has been linked to variations in the activation of ventral anterior cingulate cortex during reward learning (Eckstrand et al., 2019), as well as to the functioning of the salience network in response to adversity (McLaughlin et al., 2019). In addition, lower connectivity between the ventral anterior cingulate and reward-related regions including the prefrontal cortex and insula during reward prediction errors has been associated with greater anxiety and anxious arousal in young adults with trauma (Eckstrand et al., 2019), suggesting a common reward-related substrate between trauma and anxiety symptoms. Despite these insights, findings on striatal responses to reward are inconsistent, with some studies reporting increased activation (Hendrikse et al., 2022) and others showing diminished activation (Boecker et al., 2014; Hanson et al., 2015). This highlights the complex impact of trauma on reward-related brain systems and evidence that the ventral striatum is particularly vulnerable to maltreatment during early development, impacting its response to rewards (Teicher et al., 2016).

Despite the robust association between trauma exposure and anxiety symptoms (Elmore and Crouch, 2020; Mersky et al., 2013), not all individuals who experience trauma go on to develop anxiety symptoms (Panagou and MacBeth, 2022). The reasons why some youth with trauma develop persistent anxiety while others do not remain unclear. Researchers have implicated alterations in neural reward systems (e.g., striatum/putamen, amygdala, and prefrontal cortex) as a potential biomarker for both trauma exposure and anxiety symptoms (Anderson et al., 2023; Liuzzi et al., 2024) suggesting that differences in reward-related neural connectivity may contribute to individual variability in anxiety risk. For instance, these differing patterns of brain activity may differentiate adolescents with both higher trauma exposure and anxiety from those with higher trauma but lower anxiety, as well as those with anxiety in the absence of significant trauma exposure. Exploring the more nuanced relationships between trauma and anxiety has the potential to facilitate a better understanding of how trauma exposure moderates anxiety symptoms and thus provide insights into potential neurobiological markers of different anxiety pathways. This study aims to investigate how amygdala and ventral striatum connectivity may be associated with anxiety symptoms in adolescents with varying levels of trauma exposure. We expect that amygdala and ventral striatum connectivity will moderate anxiety risk, such that adolescents with higher anxiety symptoms will show distinct patterns of reward system connectivity, depending on their trauma exposure level. By identifying neural patterns linked to anxiety risk, this work contributes to a growing body of research that seeks to clarify the mechanisms underlying anxiety development and inform future studies on potential intervention targets. Such mechanisms may lay the foundation for designing novel hypothesis-driven medical and behavioral interventions.

## 2. Materials and methods

### 2.1. Participants

Forty-eight treatment seeking participants were recruited to participate in one of two cognitive behavioral therapy (CBT) studies in the San Diego area (*n* = 31 for the trauma-focused study [Sample A] and *n* = 17 for the anxiety- and depression-focused study [Sample B]; both samples evidenced large variation of trauma presented in the appendix [Fig. A.1] and anxiety [Fig. A.2]) (Yang et al., 2021). Data in the present paper were from the baseline scan of these youths, prior to treatment, or from the control arm of the study. Participants were 11–19 years old (M = 14.92, SD = 1.88) with varying levels of trauma exposure, and completed a child-friendly monetary incentive delay task prior to therapy in order to elicit neural activity in the reward processing circuits during fMRI acquisition. Exclusion criteria included magnetic resonance imaging (MRI) contraindications (e.g. orthodontic braces, metal implants, claustrophobia, weight over 300 lbs.), major medical problems with clear impact on the central nervous system, or participants not able to provide assent due to lack of sufficient understanding of study procedures (assessed by a qualified research team member). Of the 48 participants, data from individuals were excluded due to an incomplete MRI scan (*n* = 2), a corrupted dataset due to technical error (n = 1), and incomplete scores from measures of interest (n = 1). The analytic sample thus included *N* = 44 participants; sociodemographic characteristics are presented in Table 1. Sociodemographic characteristics separated by Sample A and Sample B are presented in Appendix (Table A.1).

Parents of minor participants gave written permission while minors gave written assent, and participants 18 years and over gave written informed consent. Study procedures and consent forms were approved by the University of California San Diego Institutional Review Board and accepted by joint agreement by the San Diego State University Institutional Review Board.

### 2.2. Measures

#### 2.2.1. Trauma exposure

History of trauma exposure was measured using the *Childhood Trauma Questionnaire* [CTQ] (Bernstein and Fink, 1998). CTQ is a 28-item self-report measurement assessing the severity and frequency of five subscales of childhood trauma, including emotional, physical, and sexual abuse; and emotional and physical neglect. Five items are measured per subscale of trauma on a 5-point Likert scale ranging from “1 = none to minimal” to “5 = severe to extreme,” and 3 items measure minimization/denial. The total self-report scores were used for the analysis, which encapsulates the sum of the scores of all five subscales (25 items; α =0.85). When compared with therapist-rated maltreatment, the CTQ demonstrates high convergent and discriminant validity (Bernstein et al., 1997) as well as very good internal consistency in a recent meta-analysis (Badenes-Ribera et al., 2024). Higher trauma scores were defined as scores reaching one standard deviation above the mean and larger, while lower trauma scores were one standard deviation below the mean and smaller.

#### 2.2.2. Anxiety symptoms

Child anxiety symptoms were measured using child reported scores on the *Screen for Child Anxiety Related Emotional Disorders - Child Version* [SCARED-C] (Birmaher et al., 1997). SCARED-C is a 41-item self-report measurement assessing five subscales of anxiety disorders, including panic/somatic symptoms, generalized anxiety, separation anxiety, social phobia and school avoidance, over the past three months in children aged 8–18 years old, as well as an overall total score, which was used in this study. Items on the measurement are on a 3-point Likert scale ranging from “0 = Not True or Hardly Ever True” to “2 = Very True or Often True” (41 items; α =0.82). SCARED-C showed good internal consistency and discriminative validity in clinically referred children and adolescents (Monga et al., 2000). Additionally, the SCARED-C total score showed high internal consistency in outpatient children (Birmaher et al., 1997). Higher anxiety scores were defined as scores reaching one standard deviation above the mean and larger, while lower anxiety scores were one standard deviation below the mean and smaller.

#### 2.2.3. fMRI data acquisition

Depending on the recruitment source, adolescents were scanned on two scanners. Data acquisition parameters and participant procedures did not vary significantly between the two recruitment sources and to examine any impact on results, post-hoc analyses included scanner and recruitment source as a covariate. Multiband procedures were utilized to increase spatial and temporal resolution. The subjects in Sample A were scanned using a 3 Tesla Siemens Magnetom Prisma with a 30-channel head coil, whereas Sample B was scanned using a 3 Tesla General Electric scanner with a 32-channel head coil. T2 blood oxygen level dependent (BOLD) images were acquired using a 3D multiband EPI pulse sequence across 3 runs for both scanners. For whole-brain coverage, there were 60 interleaved sagittal slices approximately parallel to the AC-PC line (voxel size = 2.4 × 2.4 × 2.4 mm, 358 image volumes per run, matrix size = 104 × 104 × 60, acceleration factor = 6, TR = 800 ms, TE = 30.8, flip angle = 52°, FOV = 216 mm, Sample A; voxel size = 2 × 2 × 2 mm, 370 image volumes per run, matrix size = 104 × 104 × 60, acceleration factor = 6, TR = 800 ms, TE = 29 ms, flip angle = 52°, FOV = 208 mm, Sample B). Anatomical images with prospective motion correction (T2-weighted MPRAGE PROMO) were obtained for anatomical localization and spatial normalization (429 sagittal slices, flip angle = 9°, matrix size = 256 × 256 × 176, FOV = 256 mm, voxel size = 1 × 1 × 1 mm, Sample A; 256 sagittal slices, flip angle = 8°, matrix size = 256 × 256 × 176, FOV = 256 mm, voxel size = 1 × 1 × 1 mm, Sample B).

#### 2.2.4. Reward processing task

Participants performed the Piñata task, a previously validated child-friendly monetary incentive delay task (Fig. A.1) (Dougherty et al., 2018; Helfinstein et al., 2013; Knutson et al., 2000), to assess brain activity associated with reward anticipation and feedback during fMRI acquisition. In the Piñata task, participants were instructed to hit a target (the piñata) by pressing a button when the target is in the middle of the screen in order to receive stars. Participants were also instructed that the stars gained would be exchanged for money (up to $15) at the end of the session.

Each trial began with an anticipation period that involved a cue (a piñata at the top of the screen; 2000 ms) that signaled to the participant whether or not they could earn a reward for that trial (50 % reward condition of piñata with stars, 50 % no-reward condition of piñata without stars) and a variable delay period (2500–5500 ms) where participants waited to hit the target. The target then appeared in the middle of the screen for the participant to attempt to hit and, after another short delay, the feedback period showed the result of either hitting or missing the target. In the event that the participant hit the piñata within the allotted time (500 ms, adjusted ±50 ms in real-time based on performance), the piñata broke (1500 ms) and stars fell into their basket (1500 ms) followed by a basket full of stars (1500 ms) in reward conditions. In no-reward conditions, the piñata broke, and an empty basket was shown (1500 ms). If the participant missed the target, the piñata flew away (1500 ms) and an empty basket was shown no matter the reward condition. A total of 60 trials (30 reward trials, 30 no-reward trials) were conducted among three task runs, each being 4 min 52 s. Participants viewed the task via a mirror attached to the head coil angled to view a screen with the task projected onto it.

### 2.3. Statistical analysis

#### 2.3.1. fMRI data preprocessing and first level analysis

Analysis of Functional NeuroImages (AFNI; https://afni.nimh.nih.gov/afni) was utilized for preprocessing protocols, which included functional image realignment, slice-time correction, spatial smoothing of 4 mm FWHM, and non-linear registration for spatial standardization to the Talairach template (Talairach and Tournoux, 1988). Image volume pairs with framewise displacement >1 mm was censored from individual level analysis. All participants evidenced mean framewise displacement (head motion) ≤ 0.30 mm. Voxel-wise connectivity images were created for both anticipation and feedback periods by calculating amygdala and ventral striatum connectivity (left and right separately) with the rest of the brain. These seed regions were chosen due to involvement of the ventral striatum (Fareri et al., 2015; Galván, 2013) and amygdala (Ernst, 2014; Ernst et al., 2005; Richard et al., 2013) in reward processing, in addition to involvement in previous literature regarding the relationship between trauma (Boecker et al., 2014; Hendrikse et al., 2022) and anxiety (Lahat et al., 2016; Sequeira et al., 2022; Xie et al., 2021) to reward processing. The seed regions were defined based on the Talairach atlas in AFNI (left amygdala = 1288 mm^3^; right amygdala = 1280 mm^3^; left ventral striatum = 136 mm^3^; right ventral striatum = 168 mm^3^).

#### 2.3.2. Second level analysis

To examine trauma exposure as a moderator for the relationship between anxiety symptoms and neural reward processing, we performed a repeated measures ANCOVA model using AFNI’s 3dMVM (Chen et al., 2014) with functional connectivity during reward anticipation and feedback. For reward anticipation, we modeled reward condition (reward vs. no-reward) as the within-subject factor and trauma exposure and anxiety as the between-subject variables. For reward feedback, performance (hit vs. miss) was included as an additional within-subject factor. Our contrasts of interest were the trauma and anxiety interaction effects (i.e., Trauma × Anxiety, Trauma × Anxiety × Condition for anticipation; Trauma × Anxiety, Trauma × Anxiety × Condition, and Trauma × Anxiety × Performance for feedback). Models were run separately for left and right amygdala and ventral striatum seeds. We used AFNI’s 3dClustSim to calculate the cluster thresholds for each analysis, using the mixed-model spatial autocorrelation function (−acf) and the NN1 bi-sided option, and resulted in a cluster threshold ranging from k = 58–61 depending on the seed region and task period. Across results, the cluster-level alpha was 0.05 with a conservative height threshold of *p* < 0.005.

Additionally, post-hoc analyses examined additional factors that may have impacted results: age, biological sex, recruitment source, average censored motion, and comorbid depression symptoms. Values were extracted and averaged for each significant connectivity cluster and exported to RStudio v.2024.04.1 (RStudio Team, 2020). For each cluster, the analyses were recreated with each of these factors added to the model as a covariate to determine whether the cluster was still significant after adjusting for the covariate. Packages to conduct the analyses included lme4 (Bates et al., 2015), lmertest (Kuznetsova et al., 2017), matrix (Bates et al., 2024), and car (Fox and Weisberg, 2019).

## 3. Results

Significant clusters in contrasts of interest are reported here. Full results are available in the Appendix (Table A.2).

### 3.1. Anticipation period

#### 3.1.1. Trauma × anxiety × condition

We observed significant Trauma × Anxiety × Condition interactions for multiple regions, including bilateral amygdala and left ventral striatum connectivity with multiple prefrontal, temporal, and occipital regions (Table 2, Fig. 1.A.2). The pattern of the interactions was similar in all clusters: Adolescents with lower trauma and lower anxiety levels and adolescents with higher trauma and lower anxiety levels were marked by decreased connectivity in the reward vs. no reward conditions. By contrast, adolescents with lower trauma and higher anxiety and adolescents with higher trauma and higher anxiety levels exhibited increased connectivity in reward vs. no reward conditions.

#### 3.1.2. Trauma × anxiety

We observed significant Trauma × Anxiety interactions for multiple regions, including bilateral amygdala connectivity with prefrontal and temporal regions, and left ventral striatal connectivity with widespread prefrontal/frontal and occipital regions (Table 2, Fig. 1.A.1). The pattern of the interactions was similar in all clusters: across reward and no reward conditions, adolescents with lower trauma and lower anxiety showed increased connectivity compared to adolescents with lower trauma and higher anxiety. By contrast, adolescents with higher trauma and lower anxiety had decreased connectivity compared to adolescents with higher trauma and higher anxiety.

### 3.2. Feedback period

#### 3.2.1. Trauma × anxiety × condition × response

We observed significant Trauma × Anxiety × Response × Condition interactions for multiple regions, including left amygdala connectivity with temporal regions and insula, bilateral ventral striatal connectivity with temporal regions, and left ventral striatum connectivity with lateral prefrontal and occipital regions (Table 3, Fig. 1.B.4). The pattern of the interactions was similar in all clusters: adolescents with lower trauma and lower anxiety exhibited decreased connectivity during hits vs. misses in the reward condition but increased connectivity during hits vs. misses in the no reward condition. However, adolescents with lower trauma and higher anxiety exhibited decreased connectivity during hit vs. miss trials, across both reward and no reward conditions. Adolescents with higher trauma and lower anxiety exhibited increased connectivity during hits vs. misses in reward conditions and exhibited decreased connectivity during hits vs. misses in no reward conditions. Adolescents with higher trauma and higher anxiety exhibited decreased connectivity during hit vs. miss trials across reward and no reward conditions.

#### 3.2.2. Trauma × anxiety × condition

We observed significant Trauma × Anxiety × Condition interactions for multiple regions, including bilateral amygdala connectivity with occipital regions, right amygdala connectivity with prefrontal and frontal regions, bilateral ventral striatum connectivity with lateral prefrontal, frontal, and occipital regions, and left ventral striatum connectivity with temporal regions and caudate (Table 3, Fig. 1.B.2). The pattern of the interactions was similar in all clusters: Adolescents with lower trauma and lower anxiety levels and adolescents with higher trauma and lower anxiety levels were marked by decreased connectivity in the reward vs. no reward conditions. By contrast, adolescents with lower trauma and higher anxiety and adolescents with higher trauma and higher anxiety levels exhibited increased connectivity in reward vs. no reward conditions.

#### 3.2.3. Trauma × anxiety × response

We observed significant Trauma × Anxiety × Response interactions for multiple regions, including bilateral amygdala connectivity with frontal and lateral prefrontal regions, left amygdala connectivity with parietal and cingulate cortex regions, right amygdala connectivity with temporal regions, and left ventral striatum connectivity with insula, prefrontal and parietal regions (Table 3, Fig. 1.B.3). The pattern of the interactions was similar in all clusters: adolescents with lower trauma and lower anxiety and adolescents with higher trauma and higher anxiety exhibited decreased connectivity during hit vs. miss trials. By contrast, adolescents with lower trauma and higher anxiety and adolescents with higher trauma and lower anxiety exhibited increased connectivity during hit vs. miss trials.

#### 3.2.4. Trauma × anxiety

We observed significant Trauma × Anxiety interactions for multiple regions, including bilateral amygdala connectivity with lateral prefrontal regions, left amygdala connectivity with cingulate cortex, right amygdala connectivity with temporal and parietal regions, and left ventral striatum connectivity with lateral prefrontal and occipital regions (Table 3, Fig. 1.B.1). The pattern of the interactions was similar in all clusters: across reward and no reward conditions, adolescents with lower trauma and lower anxiety showed increased connectivity compared to adolescents with lower trauma and higher anxiety. By contrast, adolescents with higher trauma and lower anxiety had decreased connectivity compared to adolescents with higher trauma and higher anxiety.

### 3.3. Additional analyses

Age, biological sex, recruitment source, average censored motion, and depression were each entered as a covariate in our GLM (model) one at a time to examine their impact on our results. Post-hoc analysis of these variables revealed no impact on the significance of any of our clusters.

## 4. Discussion

One of the major conundrums that faces psychiatric medicine is the multifinality of outcomes after exposure to psychiatric “pathogens” such as trauma. Yet, the silver lining of this puzzle (“why do some youths with trauma develop anxiety while others do not?”) is that it presents important opportunities to identify trauma-related and non-trauma-related pathways to anxiety. Understanding the neurobiological mechanisms that differentiate trauma-related pathways to psychopathology is crucial for refining models of anxiety development. This study aimed to clarify these mechanisms within a sample of adolescents with varying trauma exposure, identifying distinct neural subtypes that contribute to our understanding of trauma-related anxiety and inform future studies on potential biomarkers. By mapping the reward-related neural alterations associated with different trauma and anxiety profiles, this study addresses a critical gap in how reward processing differentiates trauma-exposed youth who develop anxiety from those with alternative trajectories.

Our findings suggest that alterations in reward processing may play a critical role in understanding the unique neural subtypes of adolescents with varying levels of trauma and anxiety. Using a reward task, we identified four distinct neural subtypes that reflect different profiles: higher anxiety/higher trauma (indicative of a trauma-related anxiety phenotype), higher trauma/lower anxiety (non-anxiety development), higher anxiety/lower trauma (non-trauma-related anxiety), and lower anxiety/lower trauma (representing typical development). Importantly, however, our dimensional analytic approach preserved natural variation in the severity of trauma and anxiety symptoms, consistent with RDoC principles (Cuthbert and Insel, 2013). This delineation suggests potential links between trauma and anxiety via altered reward processing, though the literature has been less consistent in showing reward associations for anxiety alone. While our findings support a potential link between trauma and anxiety via altered reward processing, we acknowledge that trauma is not the sole etiological pathway to anxiety. Anxiety can arise through multiple developmental trajectories, including non-trauma-related pathways, and comorbid presentations are common. Our results support the need for more research to thoroughly explore other contributing factors of anxiety development, like differing psychopathologies, to uncover how altered reward processing interacts with these mechanisms to shape anxiety outcomes.

Virtually all of the regions implicated in the different types of profiles in our study are associated with emotion regulation and reward/salience, including clusters across the prefrontal cortex, amygdala, and ventral striatum, reinforcing the importance of these neural substrates for anxiety development risk. We observed that these differences among the subtypes were driven by conditions involving a potential reward (anticipating as well as receiving feedback about a reward), i.e., in amygdala-prefrontal/insula and ventral striatum-prefrontal circuits. The Trauma × Anxiety × Condition × Performance contrast revealed a distinct insula pattern, suggesting performance-based feedback may uniquely influence connectivity. The insula plays a key role in interoception, salience detection, and performance monitoring, and its activity may differ when tracking reward presence vs. performance outcomes (Menon and Uddin, 2010). This finding suggests that performance-related salience, rather than reward availability alone, may drive insula connectivity changes. Further research should explore how performance modulates reward processing in trauma-exposed and anxious youth.

Interestingly, such differences among subtypes were also driven by the “no reward” condition, when no money was at stake, i.e., in ventral striatum-ventrolateral prefrontal circuits. These regions are critical for processing both reward and inhibitory control, yet even when no reward was presented, these circuits showed pronounced alterations in trauma-exposed individuals, highlighting the pervasive nature of the neural dysfunction that transcends motivation levels. Historically, the no reward condition was designed to be a neutral control in early versions of the monetary incentive delay task, but as this task has been applied to clinical samples it is becoming increasingly clear that neural mechanisms of psychopathology are apparent when it “shouldn’t matter”, i.e., when no reward is at stake (Dougherty et al., 2018; Parker et al., 2025). This suggests that altered reward processing in trauma-exposed individuals may not solely be a product of motivation but could reflect more fundamental changes. For example, the higher trauma/higher anxiety group likely demonstrates heightened sensitivity to both rewarding and aversive cues, which may overwhelm the brain’s reward processing system. In contrast, the higher trauma/lower anxiety group may exhibit reduced sensitivity to motivational cues, reflecting a different form of emotional and neural dysregulation. These findings emphasize the need for further research into how trauma and anxiety interact to shape the functioning of brain regions involved in motivation, emotion regulation, and cognitive control, particularly in contexts where external rewards are not present. Identifying how individual differences in reward system function shape symptom development will be an important step toward refining models of psychiatric vulnerability (Dichter et al., 2012; Nusslock and Alloy, 2017).

In a similar vein, differences among the trauma-related anxiety and non-anxiety development groups in the amygdala-dorsolateral prefrontal cortex were driven by both hit and miss conditions, although the differences were much more dramatic for the miss condition, especially among those youths with higher trauma. The pronounced effects observed in the miss condition (greater connectivity in those with higher trauma and higher anxiety versus lower connectivity in those with higher trauma but lower anxiety) suggest that failing, i.e., missing a target, may serve as an evocative probe for youth with trauma histories – though youths without anxiety development may escape the anxiety effects of trauma. This highlights how trauma-exposed youth regulate emotional responses to failure. The amygdala, central to threat detection and emotional salience, interacts with the dorsolateral prefrontal cortex, which modulates top-down control. Greater connectivity in higher trauma/higher anxiety youth may reflect heightened emotional reactivity and increased cognitive effort to regulate distress, whereas lower connectivity in higher trauma/lower anxiety youth suggests a diminished need for such regulation, potentially indicating adaptive coping. Such responses may characterize the distinct neural patterns seen in different trauma-anxiety subtypes, offering a valuable framework for identifying at-risk youth and distinguishing them from those who do not develop anxiety. Overall, our findings underscore the importance of all reward contingency situations (whether there are vs. are not stakes, succeeding vs. failing) in understanding trauma- and anxiety-related alterations in reward processing.

Our study has several limitations. The relatively small sample size may limit the generalizability of our findings. While the focus of collecting this trauma- and anxiety-enriched sample was to encompass adolescents from underrepresented populations to provide an in-depth look, larger samples will be needed to improve the statistical power to detect more subtle interactions and perform replication. Additionally, we used the CTQ to assess trauma history based on childhood abuse and neglect, a crucial contribution especially given that this sensitive information is not available in consortium datasets of adolescents (e.g., Adolescent Brain and Cognitive Development [ABCD] Study, Imagen). However, the CTQ does not fully capture the full range of trauma experiences, as it does not account for other trauma types such as peer victimization, community or school violence, natural disasters, or loss of a loved one. Although our measure offers an advantage over some large prior studies by ensuring well-defined, commonly occurring trauma categories, future research could incorporate structured interviews to obtain a more comprehensive trauma profile. Lastly, the cross-sectional design of our study limits our ability to make causal inferences. While we observed associations among trauma, anxiety, and reward processing, we cannot determine whether trauma exposure and anxiety symptoms lead to the observed neural changes or if certain pre-existing neural patterns increase susceptibility to anxiety following trauma. A longitudinal design would allow researchers to make stronger inferences on whether these neural patterns emerge as a direct consequence of trauma or reflect underlying vulnerabilities.

## 5. Conclusions

This study contributes to understanding how trauma and anxiety interact within the adolescent brain’s reward systems, illuminating distinct neural pathways of risk for anxiety development. Our findings highlight the importance of reward-related neural alterations, particularly within the prefrontal cortex, amygdala, and ventral striatum, in distinguishing those more likely to develop anxiety. By delineating anxiety vulnerability subtypes, these results contribute to a framework for future research aimed at identifying neurobiological markers of risk. This step forward lays the groundwork to work toward developing targeted, neurobiologically informed interventions to reduce the likelihood of anxiety following trauma.

## Supplementary Material

supplemental material

## Figures and Tables

**Fig. 1.A.**
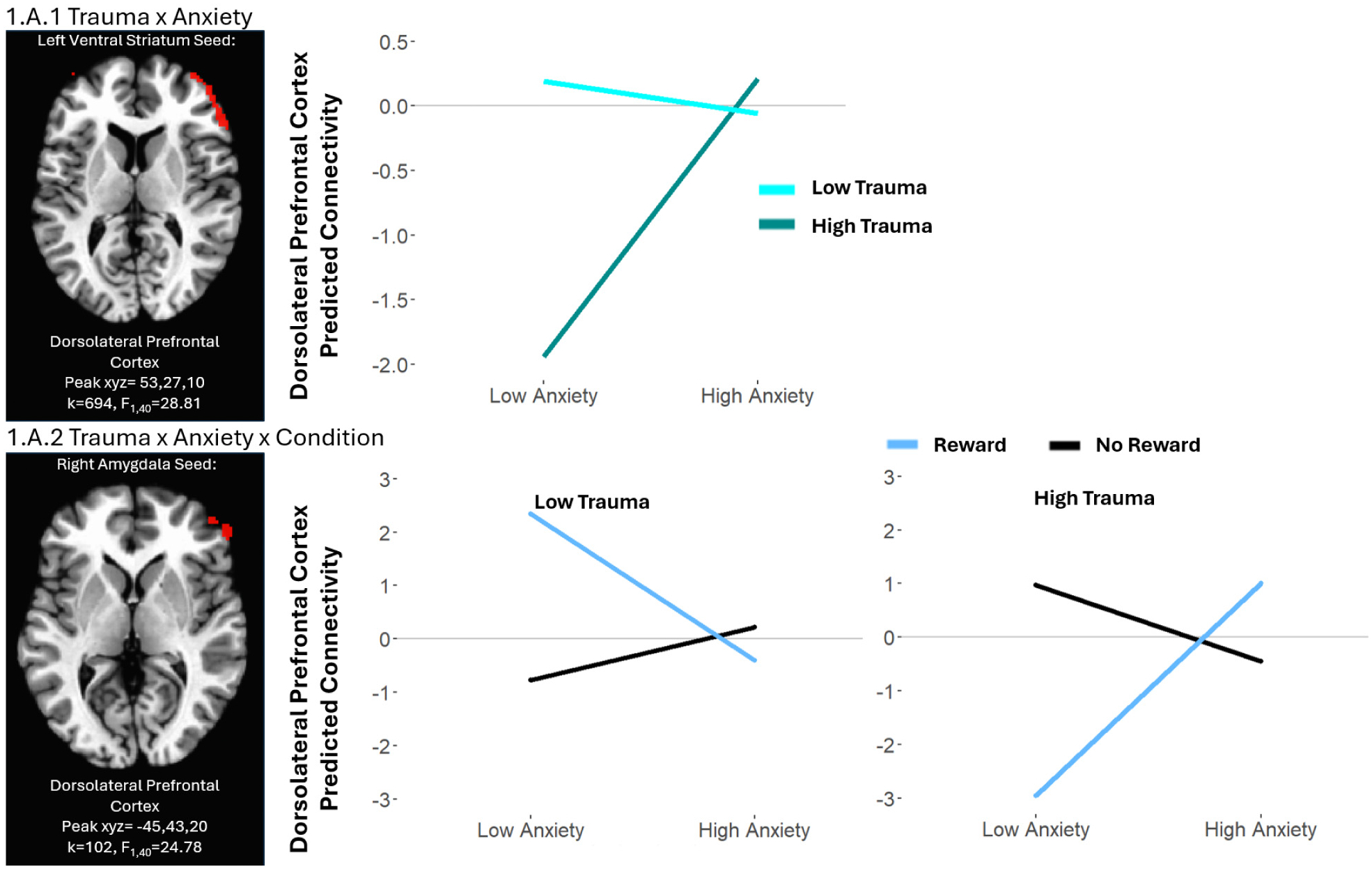
The moderating effects of trauma on anxiety-related whole-brain connectivity based on amygdala and ventral striatum seed regions during reward anticipation. For illustrative purposes, all graphs represent predicted connectivity values for indicated clusters based on the high and low trauma and anxiety scores (high and low were defined as +/− 1 standard deviation from the mean). Selected significant clusters are shown in brain pictures with a threshold set at whole-brain adjusted p < 0.05. When a contrast comprises several regions with similar patterns, only one cluster is presented as an example.

**Fig. 1.B.**
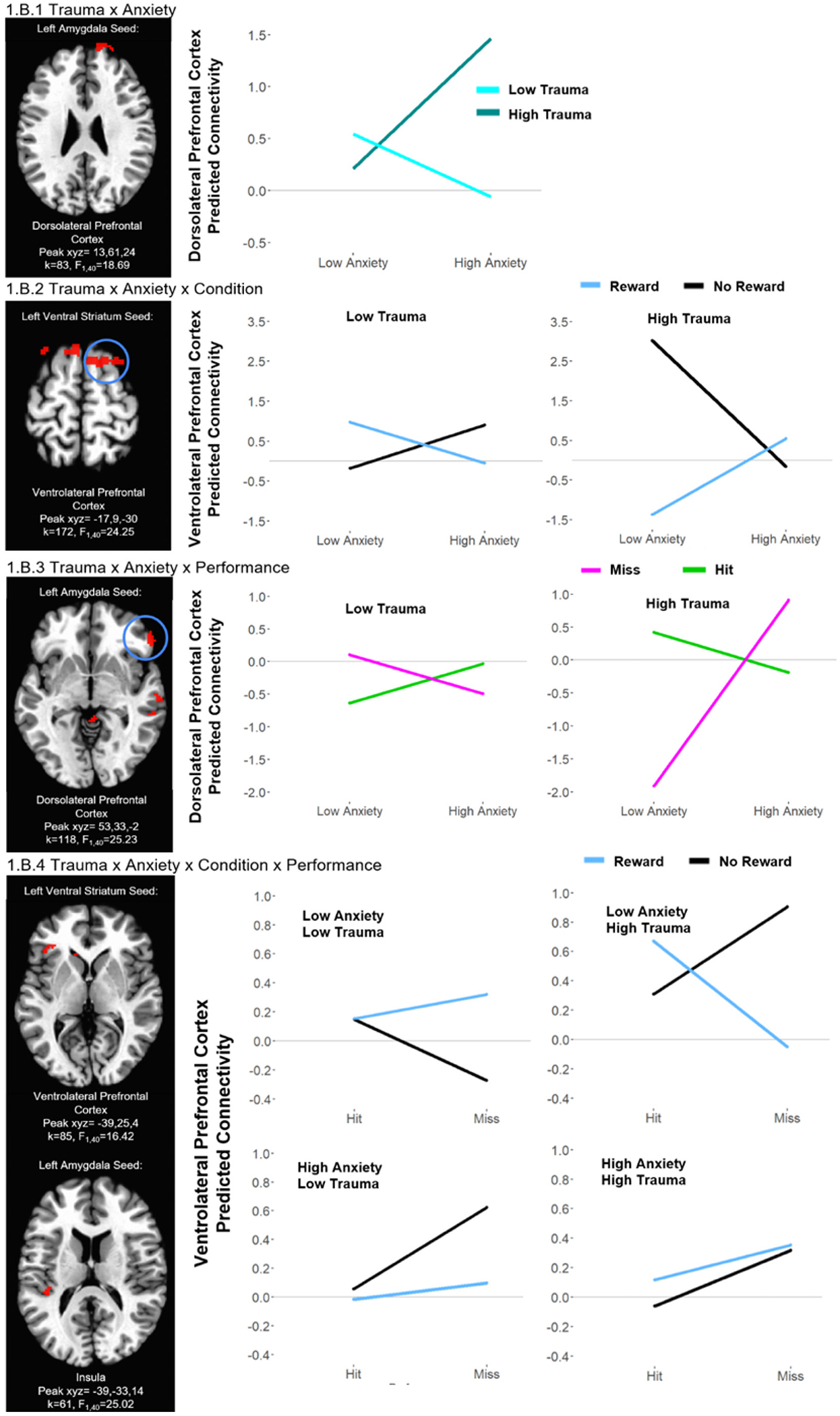
The moderating effects of trauma on anxiety-related whole-brain connectivity based on amygdala and ventral striatum seed regions during performance feedback. For illustrative purposes, all graphs represent predicted connectivity values for indicated clusters based on the high and low trauma and anxiety scores (high and low were defined as +/− 1 standard deviation from the mean). Selected significant clusters are shown in brain pictures with a threshold set at whole-brain adjusted *p* < 0.05. When a contrast comprises several regions with similar patterns, only one cluster is presented as an example.

**Table 1:** Demographic characteristics of the sample (N = 44). Summary of participant demographics, including age, gender, study group assignment, and other relevant characteristics.

	Mean	Standard deviation	Range
Age (years)	14.87	1.88	11.92–19.44
CTQ total - trauma exposure	37.43	10.10	26–67
SCARED - anxiety symptoms	23.46	16.07	0–58
MFQ - depression symptoms	11.65	11.56	0–40
		N	Percentage
Gender			
Female		24	54.5 %
Male		20	45.5 %
Race/ethnicity			
African American		4	9.1 %
Asian/Pacific Islander		2	4.5 %
White		6	13.6 %
Hispanic		23	52.3 %
Biracial		8	18.2 %
Other/unknown		1	2.3 %

*Note*. CTQ = Child Trauma Questionnaire; SCARED = Screen for Child Anxiety Related Disorders; MFQ = Mood and Feelings Questionnaire.

**Table 2:** Model results from connectivity analysis during reward anticipation Significant cluster results showing the associations between trauma exposure, anxiety symptoms, and neural connectivity during the anticipation phase of monetary reward task.

Left Amygdala Seed					
***Trauma × Anxiety***					
**k**	**F (df = 1,41)**	**x**	**y**	**z**	**Region**
158	26.3	−45	−31	56	Left Postcentral Gyrus
103	17.3	53	35	14	Right Middle Frontal Gyrus, Right Inferior Frontal Gyrus
99	22.4	37	11	−32	Right Superior Temporal Gyrus
***Trauma × Anxiety × Condition***					
80	16.8	17	55	34	Right Superior Frontal Gyrus
Right Amygdala Seed					
***Trauma × Anxiety***					
**k**	**F (df = 1,41)**	**x**	**y**	**z**	**Region**
645	29.4	41	51	18	Right Inferior Frontal Gyrus, Right Middle Frontal Gyrus
123	18.0	37	49	26	Right Superior Frontal Gyrus, Right Middle Frontal Gyrus
89	25.8	−41	−21	−22	Left Inferior Temporal Gyrus, Left Parahippocampal Gyrus
***Trauma × Anxiety × Condition***					
164	23.1	63	−23	−22	Right Inferior Temporal Gyrus, Right Fusiform Gyrus
139	26.1	47	1	−12	Right Middle Temporal Gyrus, Right Superior Temporal Gyrus
102	24.8	−45	43	20	Left Middle Frontal Gyrus
92	19.5	55	−1	2	Right Superior Temporal Gyrus
91	18.7	35	−77	−4	Right Middle Occipital Lobe
88	29.4	−23	59	24	Left Superior Frontal Gyrus
81	30.4	51	19	40	Right Middle Frontal Gyrus
66	18.4	43	−9	12	Right Insula
Left Ventral Striatum Seed					
***Trauma × Anxiety***					
**k**	**F (df = 1,41)**	**x**	**y**	**z**	**Region**
694	28.8	53	27	10	Right Middle Frontal Gyrus, Right Inferior Frontal Gyrus, Superior Frontal Gyrus
122	19.7	−43	47	18	Left Middle Frontal Gyrus, Left Superior Frontal Gyrus
122	21.1	−37	−15	62	Precentral Gyrus
90	29.1	−29	35	48	Left Middle Frontal Gyrus
60	22.6	13	−3	44	Right Cingulate Gyrus
59	33.1	29	−75	−28	Right Uvula, Right Pyramis
59	22.5	−31	−79	−12	Left Fusiform Gyrus
***Trauma × Anxiety × Condition***					
234	33.2	51	1	−10	Right Superior Temporal Gyrus, Right Middle Temporal Gyrus
178	29.2	−3	−47	0	Left Posterior Cingulate
105	15.1	−11	−101	8	Left Cuneus
75	19.7	51	17	36	Right Middle Frontal Gyrus
61	15.2	51	27	26	Right Middle Frontal Gyrus

**Table 3:** Model results from connectivity analysis during performance feedback Significant cluster results showing the associations between trauma exposure, anxiety symptoms, and neural connectivity during the performance feedback phase of monetary reward task.

Left Amygdala Seed					
***Trauma × Anxiety***					
**k**	**F (df = 1,41)**	**x**	**y**	**z**	**Region**
83	18.7	13	61	24	Right Superior Frontal Gyrus
67	29.9	−5	−47	12	Left Posterior Cingulate
***Trauma × Anxiety × Condition***					
100	21.7	13	−99	4	Right Cuneus, Right Middle Occipital Gyrus
***Trauma × Anxiety × Performance***					
331	48.8	−43	−43	32	Left Supramarginal Gyrus
259	34.2	47	−59	20	Right Superior Temporal Gyrus
171	35.7	13	45	42	Right Superior Frontal Gyrus
149	28.3	−5	−41	6	Left Posterior Cingulate
128	21.7	−5	35	50	Left Superior Frontal Gyrus
118	25.2	53	33	−2	Right Inferior Frontal Gyrus
95	28.4	33	15	48	Right Middle Frontal Gyrus, Right Superior Frontal Gyrus
92	22.7	57	−33	2	Right Middle Temporal Gyrus
77	21.6	−9	59	30	Left Superior Frontal Gyrus
72	25.6	13	63	24	Right Superior Frontal Gyrus
64	21.2	1	−59	28	Right Cingulate Gyrus, Right Precuneus
***Trauma × Anxiety × Condition × Performance***					
121	35.0	−45	−69	36	Left Angular Gyrus
65	23.4	9	−77	−10	Right Lingual Gyrus
61	25.0	−39	−33	14	Left Superior Temporal Gyrus, Insula
Right Amygdala Seed					
***Trauma × Anxiety***					
**k**	**F (df = 1,41)**	**x**	**y**	**z**	**Region**
168	25.5	15	63	22	Right Superior Frontal Gyrus
90	19.8	−39	−9	−24	Left Fusiform Gyrus, Left Inferior Temporal Gyrus, Left Middle Temporal Gyrus
67	29.4	−47	−31	56	Left Postcentral Gyrus
***Trauma × Anxiety × Condition***					
215	18.1	23	−49	−34	Right Cerebellum
202	25.9	−23	−65	−30	Bilateral Pyramis, Left Cerebellar Tonsil
114	15.2	−3	−99	6	Left Cuneus
106	26.7	−43	−77	14	Left Middle Occipital Gyrus
100	17.1	−25	39	44	Left Superior Frontal Gyrus
95	15.6	19	49	40	Right Superior Frontal Gyrus
70	21.1	33	−67	−34	Right Pyramis, Right Cerebellar Tonsil
66	24.6	−27	29	50	Left Superior Frontal Gyrus
***Trauma × Anxiety × Performance***					
74	22.1	47	−55	20	Right Superior Temporal Gyrus
61	20.9	23	−75	−22	Right Declive, Right Uvula
Left Ventral Striatum Seed					
***Trauma × Anxiety***					
**k**	**F (df = 1,41)**	**x**	**y**	**z**	**Region**
281	19.2	47	41	22	Right Middle Frontal Gyrus
213	17.0	53	25	24	Right Inferior Frontal Gyrus, Right Middle Frontal Gyrus
139	25.8	−17	−51	−28	Left Cerebellum Cortex
78	20.6	25	−75	−12	Right Lingula Gyrus, Right Declive
64	40.0	−41	−11	24	Left Precentral Gyrus, Left Postcentral Gyrus
***Trauma × Anxiety × Condition***					
3177	30.8	53	11	−6	Bilateral Middle Frontal Gyrus, Bilateral Superior Frontal Gyrus, Right Inferior Frontal Gyrus
415	37.9	−25	7	2	Left Lentiform Nucleus
196	18.7	−5	23	58	Right Superior Frontal Gyrus, Left Superior Frontal Gyrus
172	24.3	−17	9	−30	Left Inferior Frontal Gyrus
130	19.5	−19	−101	10	Left Cuneus, Left Middle Occipital Gyrus
120	34.3	−15	21	12	Left Caudate
83	20.9	−45	19	−16	Left Superior Temporal Gyrus
70	21.2	−31	−97	4	Right Middle Occipital Gyrus
***Trauma × Anxiety × Performance***					
398	38.8	47	9	6	Right Insula, Right Precentral Gyrus
242	27.5	−33	7	12	Left Insula, Left Precentral Gyrus
132	31.0	−57	−25	44	Left Inferior Parietal Lobule, Left Precentral Gyrus
131	33.2	−37	51	30	Left Middle Frontal Gyrus, Left Superior Frontal Gyrus
62	21.1	−49	−59	48	Left Inferior Parietal Lobule, Left Superior Parietal Lobule
***Trauma × Anxiety × Condition × Performance***					
349	32.5	−29	31	14	Left Caudate, Left Anterior Cingulate
156	23.1	−43	3	−24	Left Superior Temporal Gyrus, Left Middle Temporal Gyrus
100	26.4	39	7	−24	Right Superior Temporal Gyrus
93	31.1	23	−49	−10	Right Parahippocampal, Right Culmen
91	26.6	−25	39	44	Left Superior Frontal Gyrus
90	21.0	−29	21	58	Left Middle Frontal Gyrus, Left Superior Frontal Gyrus
85	16.4	−39	25	4	Left Inferior Frontal Gyrus
72	18.8	−39	−11	−22	Left Fusiform Gyrus, Left Inferior Temporal Gyrus
65	19.6	−37	−51	10	Left Posterior Cingulate
Right Ventral Striatum Seed					
***Trauma × Anxiety × Condition***					
**k**	**F (df = 1,41)**	**x**	**y**	**z**	**Region**
782	30.3	19	49	40	Right Middle Frontal Gyrus, Right Superior Frontal Gyrus
228	28.1	−41	33	44	Left Middle Frontal Gyrus, Left Superior Frontal Gyrus
161	26.0	55	41	4	Right Inferior Frontal Gyrus, Right Middle Orbital Gyrus
102	23.9	−23	59	22	Left Superior Frontal Gyrus, Left Middle Frontal Gyrus
87	26.1	−21	−97	14	Left Superior Occipital Gyrus
65	17.3	−17	15	62	Left Superior Frontal Gyrus, Left Middle Frontal Gyrus
***Trauma × Anxiety × Condition × Performance***					
78	18.6	35	−5	−42	Right Inferior Temporal Gyrus
62	19.2	49	−19	−22	Right Inferior Temporal Gyrus

## Appendix A. Supplementary data

Supplementary data to this article can be found online at https://doi.org/10.1016/j.jad.2025.03.076.
