# Supplementary material for: Trauma and anxiety interactions relate to reward processing in adolescents
Source: J Affect Disord. Author manuscript; Available in PMC 2026 Mar 21. (PMC13005575; doi:10.1016/j.jad.2025.03.076)
Supplement: supplemental material [file NIHMS2154912-supplement-supplemental_material.docx]

**Appendix**

**Figure A.1. Distribution of Childhood Trauma Questionnaire Scores.**

**
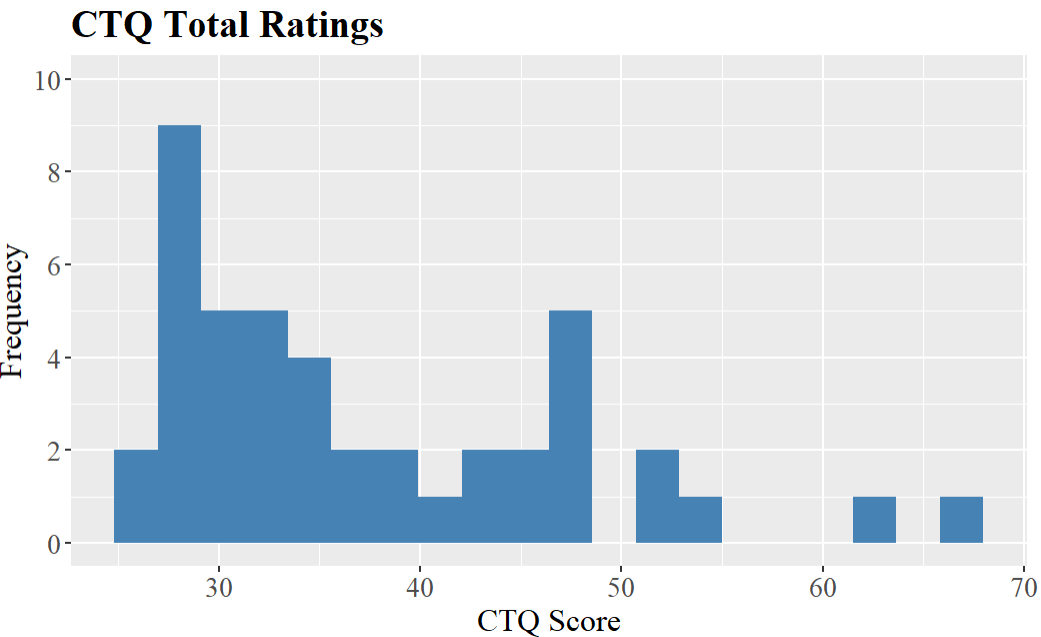
**

**Figure A.2. Distribution of Screen for Child Anxiety Related Emotional Disorders - Child Version Scores.**

**
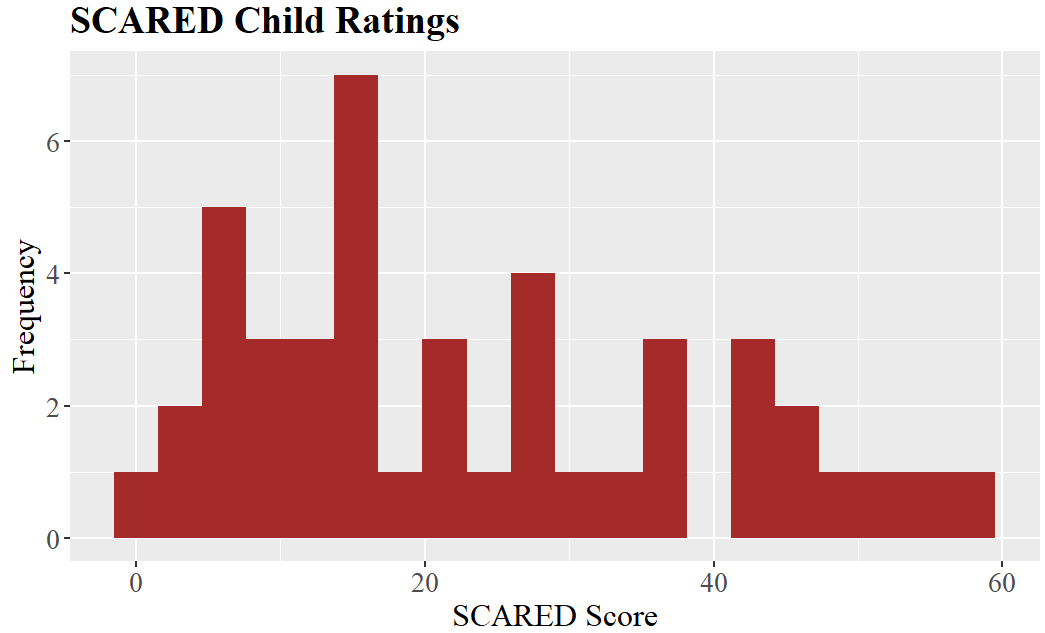
**

**Figure A.3. fMRI child-friendly monetary incentive delay task paradigm.** *Reprinted from Wiggins, J. L., Schwartz, K. T., Kryza-Lacombe, M., Spechler, P. A., Blankenship, S. L., & Dougherty, L. R. (2017). Neural reactivity to reward in school-age offspring of depressed mothers. J Affect Disord, 214, 81-88. doi:10.1016/j.jad.2017.03.020 (Wiggins et al., 2017)*

**
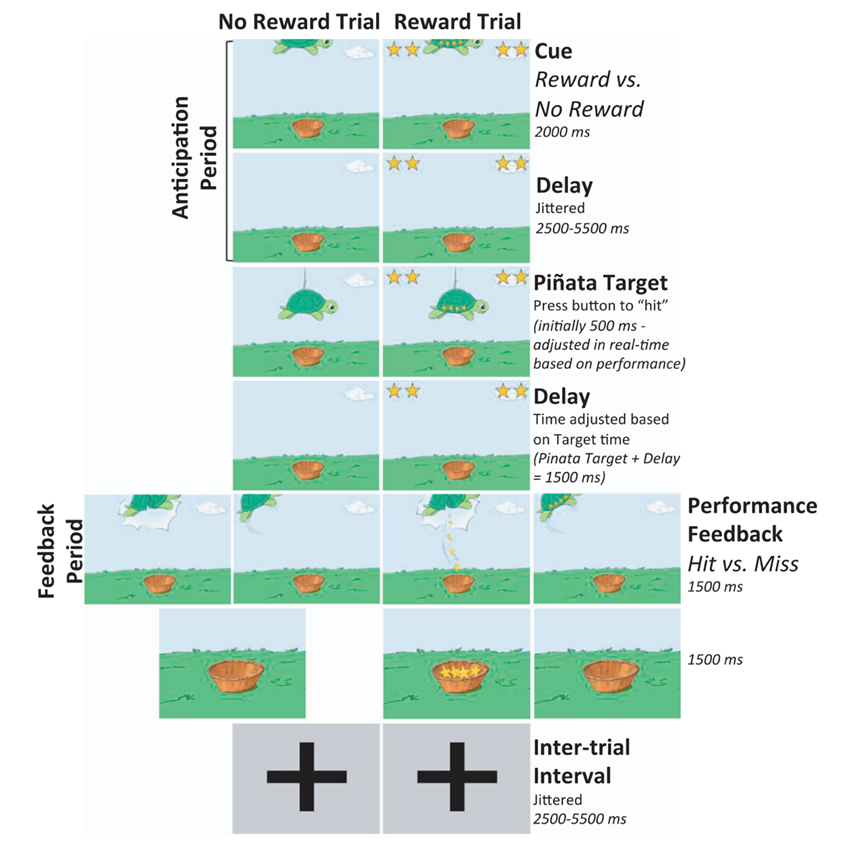
**

**Table A.1. Sociodemographic by Sample.**

| Sample A | **Mean** | **Standard Deviation** | **Range** |  |
| --- | --- | --- | --- | --- |
| Age (years) | 14.55 | 1.74 | 11.92-18.68 |  |
| CTQ Total – Trauma Exposure | 37.23 | 10.86 | 26-67 |  |
| SCARED – Anxiety Symptoms | 25.55 | 16.66 | 4-58 |  |
| MFQ – Depression Symptoms | 11.93 | 12.28 | 0-40 |  |
|  | **N** | **Percentage** |  |  |
| Gender |  |  |  |  |
| Female | 18 | 40.9% |  |  |
| Male | 13 | 29.5% |  |  |
| Race/Ethnicity |  |  |  |  |
| African American | 3 | 6.8% |  |  |
| Asian/Pacific Islander | 2 | 4.5% |  |  |
| White | 0 | 0.0% |  |  |
| Hispanic | 22 | 50.0% |  |  |
| Biracial | 4 | 9.1% |  |  |
| Other/Unknown | 0 | 0.0% |  |  |
| Sample B (n=13) | | **Mean** | **Standard Deviation** | **Range** |
| Age (years) | | 15.64 | 2.05 | 12.39-19.44 |
| CTQ Total – Trauma Exposure | | 37.92 | 8.41 | 26-52 |
| SCARED – Anxiety Symptoms | | 18.46 | 13.91 | 0-49 |
| MFQ – Depression Symptoms | | 11.00 | 10.04 | 1-35 |
|  | | **N** | **Percentage** |  |
| Gender | |  |  |  |
| Female | | 6 | 13.6% |  |
| Male | | 7 | 15.9% |  |
| Race/Ethnicity | |  |  |  |
| African American | | 1 | 2.3% |  |
| Asian/Pacific Islander | | 0 | 0.0% |  |
| White | | 6 | 13.6% |  |
| Hispanic | | 1 | 2.3% |  |
| Biracial | | 4 | 9.1% |  |
| Other/Unknown | | 1 | 2.3% |  |
| *Note.* CTQ = Child Trauma Questionnaire; SCARED = Screen for Child Anxiety Related Disorders; MFQ = Mood and Feelings Questionnaire. | | | | |

**Table A.2. Full Model Results from Connectivity Analysis**

Significant cluster results showing the associations between trauma exposure, anxiety symptoms, and neural connectivity during the anticipation and performance feedback phase of monetary reward task.

| **Left Amygdala Seed** | | | | | | |
| --- | --- | --- | --- | --- | --- | --- |
| **Reward Anticipation** | | | | | | |
| ***Trauma Main Effect*** | | | | | | |
| **k** | **F (df = 1,41)** | **x** | **y** | **z** | | **Region** |
| 556 | 38.2 | 43 | 17 | -32 | | Right Superior Temporal Gyrus, Right Cerebellar Tonsil |
| 307 | 26.0 | -49 | -27 | 42 | | Left Postcentral Gyrus, Left Inferior Parietal Lobule |
| 160 | 34.7 | -19 | -75 | -30 | | Left Pyramis, Left Tuber |
| 129 | 28.6 | 63 | -9 | 30 | | Right Precentral Gyrus |
| 119 | 30.6 | -27 | -53 | 66 | | Left Superior Parietal Lobule |
| 105 | 16.3 | 55 | 25 | 20 | | Right Middle Frontal Gyrus, Right Inferior Frontal Gyrus |
| 103 | 26.4 | 9 | -27 | -38 | | Brain Stem |
| 97 | 28.1 | 21 | -17 | 10 | | Right Putamen |
| 97 | 15.5 | -37 | -33 | 38 | | Left Inferior Parietal Lobule |
| 86 | 21.1 | 35 | 25 | 48 | | Right Middle Frontal Gyrus |
| 71 | 18.2 | 13 | -67 | -32 | | Right Uvula, Right Pyramis |
| 66 | 24.7 | 3 | -69 | -24 | | Right Declive, Right Pyramis |
| 65 | 22.7 | 19 | 49 | 34 | | Right Superior Frontal Gyrus |
| 62 | 49.4 | 47 | -61 | -32 | | Right Cerebellar Tonsil |
| ***Trauma x Anxiety*** | | | | | | |
| 158 | 26.3 | -45 | -31 | 56 | | Left Postcentral Gyrus |
| 103 | 17.3 | 53 | 35 | 14 | | Right Middle Frontal Gyrus, Right Inferior Frontal Gyrus |
| 99 | 22.4 | 37 | 11 | -32 | | Right Superior Temporal Gyrus |
| 84 | 19.4 | 27 | -39 | -36 | | Cerebellar Tonsil |
| 76 | 19.4 | -9 | -15 | -38 | | Brain Stem |
| 75 | 23.6 | -19 | -75 | -30 | | Left Pyramis, Left Tuber |
| ***Trauma x Condition*** | | | | | | |
| 91 | 18.0 | -9 | -103 | | 6 | Left Cuneus |
| 82 | 39.2 | 47 | -69 | | -34 | Right Pyramis, Right Cerebellar Tonsil |
| 69 | 24.6 | 7 | -73 | | -26 | Right Uvula, Right Pyramis |
| 58 | 16.1 | 11 | 49 | | 38 | Right Superior Frontal Gyrus |
| ***Trauma x Anxiety x Condition*** | | | | | | |
| 80 | 16.8 | 17 | 55 | | 34 | Right Superior Frontal Gyrus |
| **Performance Feedback** | | | | | | |
| ***Trauma Main Effect*** | | | | | | |
| 67 | 30.2 | 49 | 15 | | -28 | Right Superior Temporal Gyrus |
| ***Anxiety Main Effect*** | | | | | | |
| 72 | 20.2 | -35 | -63 | | 50 | Superior Parietal Lobule, Inferior Parietal Lobule |
| ***Trauma x Anxiety*** | | | | | | |
| 83 | 18.7 | 13 | 61 | | 24 | Right Superior Frontal Gyrus |
| 67 | 29.9 | -5 | -47 | | 12 | Left Posterior Cingulate |
| ***Trauma x Condition*** | | | | | | |
| 579 | 24.6 | 43 | -21 | | -32 | Right Cerebellar Tonsil |
| 300 | 26.3 | -5 | -97 | | 12 | Left Cuneus, Left Middle Occipital Gyrus |
| 257 | 24.5 | 31 | -87 | | 18 | Right Middle Occipital Gyrus, Right Cuneus |
| 175 | 26.1 | 17 | -79 | | -28 | Right Pyramis, Right Uvula |
| 102 | 25.5 | 41 | 23 | | -24 | Right Superior Temporal Gyrus |
| ***Trauma x Anxiety x Condition*** | | | | | | |
| 125 | 28.3 | -5 | -99 | | 10 | Left Cuneus |
| 100 | 21.7 | 13 | -99 | | 4 | Right Cuneus, Right Middle Occipital Gyrus |
| 73 | 26.1 | 23 | -23 | | -36 | Right Cerebellar Cortex |
| 65 | 21.6 | -25 | -15 | | -40 | Left Cerebellar Cortex |
| ***Performance Main Effect*** | | | | | | |
| 102 | 14.6 | -3 | -59 | | 54 | Left Precuneus |
| ***Trauma x Performance*** | | | | | | |
| 459 | 35.1 | 1 | -23 | | -38 | Right Cerebellar Tonsil |
| ***Anxiety x Performance*** | | | | | | |
| 75 | 19.2 | -49 | -29 | | 44 | Left Inferior Parietal Lobule, Left Postcentral Gyrus |
| ***Trauma x Anxiety x Performance*** | | | | | | |
| 331 | 48.8 | -43 | -43 | | 32 | Left Supramarginal Gyrus |
| 259 | 34.2 | 47 | -59 | | 20 | Right Superior Temporal Gyrus |
| 171 | 35.7 | 13 | 45 | | 42 | Right Superior Frontal Gyrus |
| 170 | 24.1 | 41 | -39 | | -34 | Right Cerebellar Tonsil |
| 149 | 28.3 | -5 | -41 | | 6 | Left Posterior Cingulate |
| 128 | 21.7 | -5 | 35 | | 50 | Left Superior Frontal Gyrus |
| 118 | 25.2 | 53 | 33 | | -2 | Right Inferior Frontal Gyrus |
| 95 | 28.4 | 33 | 15 | | 48 | Right Middle Frontal Gyrus, Right Superior Frontal Gyrus |
| 92 | 22.7 | 57 | -33 | | 2 | Right Middle Temporal Gyrus |
| 77 | 21.6 | -9 | 59 | | 30 | Left Superior Frontal Gyrus |
| 72 | 25.6 | 13 | 63 | | 24 | Right Superior Frontal Gyrus |
| 66 | 26.8 | 7 | -83 | | -22 | Right Pyramis, Right Uvula |
| 64 | 21.2 | 1 | -59 | | 28 | Right Cingulate Gyrus, Right Precuneus |
| ***Trauma x Condition x Performance*** | | | | | | |
| 231 | 23.1 | 37 | -39 | | -32 | Right Cerebellar Tonsil, Right Culmen |
| 180 | 24.2 | -47 | -45 | | 44 | Left Superior Parietal Lobule |
| 76 | 33.5 | 59 | -27 | | -20 | Right Inferior Temporal Gyrus |
| 66 | 29.4 | 31 | 5 | | -26 | Right Superior Temporal Gyrus |
| ***Anxiety x Condition x Performance*** | | | | | | |
| 136 | 37.0 | -47 | -45 | | 44 | Left Inferior Parietal Lobule |
| 64 | 14.9 | -41 | 31 | | 22 | Left Middle Frontal Gyrus |
| ***Trauma x Anxiety x Condition x Performance*** | | | | | | |
| 121 | 35.0 | -45 | -69 | | 36 | Left Angular Gyrus |
| 65 | 23.4 | 9 | -77 | | -10 | Right Lingual Gyrus |
| 64 | 16.7 | 49 | -37 | | -32 | Right Cumen |
| 61 | 25.0 | -39 | -33 | | 14 | Left Superior Temporal Gyrus, Insula |
| **Right Amygdala Seed** | | | | | | |
| **Reward Anticipation** | | | | | | |
| ***Trauma Main Effect*** | | | | | | |
| 452 | 28.0 | -45 | -71 | | 44 | Left Precuneus, Left Superior Parietal Lobule |
| 379 | 20.6 | 33 | 51 | | 26 | Right Middle Frontal Gyrus, Right Superior Frontal Gyrus |
| 372 | 25.4 | 63 | 3 | | 30 | Right Inferior Frontal Gyrus, Right Middle Frontal Gyrus |
| 205 | 40.9 | -41 | -21 | | -22 | Left Fusiform Gyrus, Left Inferior Temporal Gyrus |
| 118 | 16.7 | -21 | -45 | | 46 | Left Inferior Parietal Lobule, Left Precuneus |
| 90 | 23.6 | 3 | -55 | | -30 | Right Cerebellar Tonsil, Right Uvula |
| 88 | 23.0 | -35 | -9 | | 30 | Left Precentral Gyrus |
| 72 | 17.5 | 17 | -65 | | 54 | Right Precuneus |
| 70 | 17.0 | 15 | -59 | | 66 | Right Postcentral Gyrus (10%) |
| 68 | 29.7 | 5 | -69 | | -24 | Right Declive, Right Pyramis |
| 66 | 17.8 | -35 | 19 | | -28 | Left Superior Temporal Gyrus |
| 64 | 24.8 | 67 | -13 | | 22 | Right Postcentral Gyrus |
| ***Trauma x Anxiety*** | | | | | | |
| 645 | 29.4 | 41 | 51 | | 18 | Right Inferior Frontal Gyrus, Right Middle Frontal Gyrus |
| 123 | 18.0 | 37 | 49 | | 26 | Right Superior Frontal Gyrus, Right Middle Frontal Gyrus |
| 89 | 25.8 | -41 | -21 | | -22 | Left Inferior Temporal Gyrus, Left Parahippocampal Gyrus |
| ***Trauma x Condition*** | | | | | | |
| 245 | 29.0 | 33 | 9 | | -32 | Right Superior Temporal Gyrus |
| 239 | 40.3 | 45 | -23 | | -32 | Right Cerebellar Tonsil |
| 221 | 22.2 | 63 | -39 | | -18 | Right Fusiform Gyrus, Right Inferior Temporal Gyrus |
| 212 | 28.0 | 53 | 7 | | -20 | Right Middle Temporal Gyrus, Right Superior Temporal Gyrus |
| 173 | 31.1 | 31 | -95 | | -4 | Right Cuneus, Right Lingual Gyrus |
| 137 | 22.4 | 23 | 15 | | 34 | Right Middle Frontal Gyrus |
| 126 | 25.0 | 61 | -11 | | 8 | Right Superior Temporal Gyrus |
| 121 | 19.3 | 45 | -49 | | 28 | Right Superior Temporal Gyrus, Right Supramarginal Gyrus |
| 118 | 34.2 | -5 | -99 | | 10 | Left Cuneus |
| 96 | 37.5 | -51 | -57 | | -12 | Left Inferior Temporal Gyrus, Left Fusiform Gyrus |
| 88 | 29.1 | -53 | -53 | | 12 | Left Middle Temporal Gyrus |
| 86 | 29.4 | 39 | -59 | | 50 | Right Superior Parietal Lobule, Right Inferior Parietal Lobule |
| 78 | 20.3 | 27 | 27 | | 6 | Right Caudate |
| 76 | 22.2 | 5 | 57 | | 8 | Right Medial Frontal Gyrus |
| 70 | 34.0 | 35 | -75 | | -6 | Right Inferior Occipital Gyrus, Right Middle Occipital Gyrus |
| 69 | 20.1 | 37 | -17 | | 14 | Right Insula |
| 65 | 21.2 | -9 | -23 | | 2 | Left Thalamus |
| 63 | 23.3 | 1 | -67 | | -14 | Right Cerebellum Cortex |
| ***Anxiety x Condition*** | | | | | | |
| 194 | 21.2 | 41 | -59 | | 50 | Right Superior Parietal Lobule, Right Precuneus, Right Inferior Parietal Lobule |
| 126 | 21.2 | -29 | -67 | | 30 | Left Precuneus |
| 68 | 28.1 | 37 | 25 | | 22 | Right Middle Frontal Gyrus |
| ***Trauma x Anxiety x Condition*** | | | | | | |
| 164 | 23.1 | 63 | -23 | | -22 | Right Inferior Temporal Gyrus, Right Fusiform Gyrus |
| 139 | 26.1 | 47 | 1 | | -12 | Right Middle Temporal Gyrus, Right Superior Temporal Gyrus |
| 102 | 24.8 | -45 | 43 | | 20 | Left Middle Frontal Gyrus |
| 92 | 19.5 | 55 | -1 | | 2 | Right Superior Temporal Gyrus |
| 91 | 18.7 | 35 | -77 | | -4 | Right Middle Occipital Lobe |
| 88 | 29.3 | 53 | -41 | | -34 | Cerebellum |
| 88 | 29.4 | -23 | 59 | | 24 | Left Superior Frontal Gyrus |
| 81 | 30.4 | 51 | 19 | | 40 | Right Middle Frontal Gyrus |
| 72 | 26.4 | -5 | -99 | | 10 | Left Cuneus |
| 66 | 18.4 | 43 | -9 | | 12 | Right Insula |
| **Performance Feedback** | | | | | | |
| ***Trauma Main Effect*** | | | | | | |
| 388 | 33.0 | -59 | -29 | | 40 | Left Inferior Parietal Lobule, Left Supramarginal Gyrus |
| 378 | 22.5 | -7 | 65 | | 28 | Left Superior Frontal Gyrus |
| 170 | 21.4 | 31 | 37 | | 40 | Right Middle Frontal Gyrus, Right Superior Frontal Gyrus |
| 167 | 29.0 | 1 | 31 | | -18 | Right Rectal Gyrus, Right Orbital Gyrus |
| 120 | 24.8 | -7 | -97 | | 10 | Left Cuneus, Left Middle Occipital Gyrus |
| 107 | 20.0 | 19 | 47 | | 42 | Right Superior Frontal Gyrus |
| 99 | 30.5 | 51 | -63 | | 40 | Right Inferior Parietal Lobule, Right Precuneus |
| 70 | 32.6 | -35 | -53 | | 60 | Left Superior Parietal Lobule |
| 68 | 22.0 | -45 | -31 | | 58 | Left Postcentral Gyrus |
| 67 | 17.4 | -51 | 31 | | 16 | Left Inferior Frontal Gyrus, Left Middle Frontal Gyrus |
| 62 | 18.0 | 9 | -51 | | 72 | Right Postcentral Gyrus |
| ***Anxiety Main Effect*** | | | | | | |
| 60 | 17.7 | -25 | 25 | | 50 | Left Superior Frontal Gyrus |
| ***Trauma x Anxiety*** | | | | | | |
| 168 | 25.5 | 15 | 63 | | 22 | Right Superior Frontal Gyrus |
| 90 | 19.8 | -39 | -9 | | -24 | Left Fusiform Gyrus, Left Inferior Temporal Gyrus, Left Middle Temporal Gyrus |
| 67 | 29.4 | -47 | -31 | | 56 | Left Postcentral Gyrus |
| 62 | 24.2 | 7 | -79 | | -24 | Right Pyramis, Right Uvula |
| ***Condition Main Effect*** | | | | | | |
| 101 | 20.3 | -27 | 27 | | 50 | Left Superior Frontal Gyrus, Left Middle Frontal Gyrus |
| 63 | 13.2 | 45 | -37 | | 20 | Right Inferior Parietal Lobule, Right Superior Temporal Gyrus, Right Insula |
| ***Trauma x Condition*** | | | | | | |
| 1458 | 32.3 | 41 | -31 | | -32 | Bilateral Cerebellar Tonsil, Left Pyramis |
| 960 | 27.5 | 25 | 41 | | 44 | Right Superior Frontal Gyrus, Right Middle Frontal Gyrus |
| 631 | 35.1 | -19 | -99 | | -12 | Left Cuneus, Left Middle Occipital Gyrus |
| 284 | 30.9 | 27 | -95 | | -6 | Right Cuneus, Right Lingual Gyrus |
| 199 | 31.7 | 15 | -81 | | -28 | Right Pyramis, Left Declive, Right Uvula |
| 144 | 23.5 | -3 | 57 | | 34 | Left Superior Frontal Gyrus, Left Medial Frontal Gyrus |
| 101 | 30.9 | 31 | -89 | | 18 | Right Middle Occipital Gyrus |
| 90 | 23.0 | -25 | 49 | | 34 | Left Superior Frontal Gyrus |
| 75 | 25.6 | 41 | 21 | | -24 | Right Superior Temporal Gyrus |
| 73 | 17.1 | 27 | 13 | | 56 | Right Middle Frontal Gyrus, Right Superior Frontal Gyrus |
| 72 | 23.1 | -45 | 1 | | 16 | Left Precentral Gyrus, Left Inferior Gyrus |
| 70 | 24.8 | -35 | -25 | | -34 | Left Inferior Temporal Gyrus |
| 70 | 24.1 | 35 | -67 | | -34 | Right Pyramis, Right Cerebellar Tonsil |
| 67 | 24.9 | 31 | 9 | | -36 | Right Middle Temporal Gyrus, Right Superior Temporal Gyrus |
| 64 | 25.7 | -13 | 45 | | -8 | Left Medial Frontal Gyrus |
| ***Anxiety x Condition*** | | | | | | |
| 121 | 20.9 | -57 | -29 | | 34 | Left Inferior Parietal Lobule |
| 95 | 22.7 | 3 | -93 | | 4 | Right Cuneus, Right Lingual Gyrus |
| 77 | 19.2 | -23 | -27 | | 50 | Left Precentral Gyrus, Left Postcentral Gyrus |
| 66 | 30.0 | 17 | -79 | | -24 | Right Pyramis, Right Uvula |
| 61 | 23.7 | -45 | 5 | | 24 | Left Inferior Frontal Gyrus, Left Precentral Gyrus |
| ***Trauma x Anxiety x Condition*** | | | | | | |
| 215 | 18.1 | 23 | -49 | | -34 | Right Cerebellum |
| 202 | 25.9 | -23 | -65 | | -30 | Bilateral Pyramis, Left Cerebellar Tonsil |
| 114 | 15.2 | -3 | -99 | | 6 | Left Cuneus |
| 106 | 26.7 | -43 | -77 | | 14 | Left Middle Occipital Gyrus |
| 100 | 17.1 | -25 | 39 | | 44 | Left Superior Frontal Gyrus |
| 95 | 15.6 | 19 | 49 | | 40 | Right Superior Frontal Gyrus |
| 70 | 21.1 | 33 | -67 | | -34 | Right Pyramis, Right Cerebellar Tonsil |
| 66 | 24.6 | -27 | 29 | | 50 | Left Superior Frontal Gyrus |
| ***Trauma x Performance*** | | | | | | |
| 75 | 18.9 | 3 | 39 | | -18 | Bilateral Medial Frontal Gyrus, Bilateral Orbital Gyrus |
| ***Anxiety x Performance*** | | | | | | |
| 137 | 25.0 | 19 | -81 | | -44 | Right Inferior Semi-Lunar Lobule |
| ***Trauma x Anxiety x Performance*** | | | | | | |
| 74 | 22.1 | 47 | -55 | | 20 | Right Superior Temporal Gyrus |
| 61 | 20.9 | 23 | -75 | | -22 | Right Declive, Right Uvula |
| ***Condition x Performance*** | | | | | | |
| 61 | 22.6 | 47 | -19 | | -24 | Right Fusiform Gyrus, Right Inferior Temporal Gyrus |
| ***Trauma x Condition x Performance*** | | | | | | |
| 235 | 34.3 | 23 | -97 | | -8 | Right Inferior Occipital Gyrus, Right Lingual Gyrus |
| 145 | 28.3 | 11 | -75 | | -24 | Right Pyramis, Right Uvula |
| 132 | 18.7 | 43 | -49 | | -32 | Right Culmen, Right Cerebellar Tonsil |
| 127 | 22.6 | 33 | -57 | | -36 | Right Cerebellar Tonsil, Right Declive |
| 91 | 31.2 | -53 | -13 | | -28 | Left Inferior Temporal Gyrus, Left Fusiform Gyrus |
| 74 | 17.3 | 55 | 41 | | 8 | Right Inferior Frontal Gyrus, Right Middle Frontal Gyrus |
| 63 | 25.3 | -19 | -83 | | -30 | Left Pyramis, Left Tuber |
| 61 | 30.1 | 51 | -19 | | -24 | Right Fusiform Gyrus, Right Inferior Temporal Gyrus |
| ***Anxiety x Condition x Performance*** | | | | | | |
| 607 | 31.3 | -47 | 11 | | 36 | Left Middle Frontal Gyrus, Left Precentral Gyrus, Left Inferior Frontal Gyrus |
| 321 | 24.4 | -15 | 31 | | 50 | Left Superior Frontal Gyrus |
| 243 | 38.1 | -15 | -81 | | -38 | Left Inferior Semi-Lunar Lobule, Left Pyramis |
| 207 | 25.0 | 9 | -75 | | -34 | Right Pyramis, Right Uvula, Right Inferior Semi-Lunar Lobule |
| 113 | 18.6 | -63 | -27 | | 20 | Left Inferior Parietal Lobule, Left Postcentral Gyrus |
| 102 | 30.5 | -49 | -45 | | 42 | Left Inferior Parietal Lobule |
| 88 | 20.0 | -7 | -7 | | 40 | Left Cingulate Gyrus |
| 85 | 18.3 | 31 | 47 | | 0 | Right Superior Frontal Gyrus, Right Medial Frontal Gyrus, Right Middle Frontal Gyrus |
| 73 | 22.3 | -23 | -27 | | 50 | Left Precentral Gyrus, Left Postcentral Gyrus |
| 70 | 17.5 | -7 | -37 | | 32 | Bilateral Cingulate Gyrus |
| 65 | 19.4 | -43 | -35 | | 22 | Left Inferior Parietal Lobule, Left Insula |
| 62 | 39.3 | -25 | 29 | | -8 | Left Inferior Frontal Gyrus |
| 61 | 28.7 | 15 | 33 | | 28 | Right Medial Frontal Gyrus |
| **Left Ventral Striatum Seed** | | | | | | |
| **Reward Anticipation** | | | | | | |
| ***Trauma Main Effect*** | | | | | | |
| 2788 | 37.1 | -47 | 51 | | 8 | Bilateral Middle Frontal Gyrus, Bilateral Superior Frontal Gyrus, Right Inferior Frontal Gyrus |
| 275 | 41.7 | -23 | -99 | | -10 | Left Fusiform Gyrus, Left Inferior Occipital Gyrus |
| 210 | 32.6 | -15 | -97 | | 6 | Left Cuneus, Left Middle Occipital Gyrus |
| 208 | 26.3 | 65 | -15 | | 20 | Right Precentral Gyrus, Right Postcentral Gyrus |
| 187 | 28.5 | -49 | -69 | | 20 | Left Middle Temporal Gyrus, Left Superior Occipital Gyrus |
| 172 | 33.7 | -19 | 29 | | -22 | Left Inferior Frontal Gyrus, Left Rectal Gyrus |
| 168 | 31.9 | 51 | 3 | | -36 | Right Middle Temporal Gyrus |
| 162 | 25.7 | 15 | -99 | | -2 | Right Cuneus, Right Lingual Gyrus |
| 146 | 25.9 | -13 | -53 | | -32 | Left Cerebellar Tonsil |
| 123 | 31.2 | 63 | -23 | | -30 | Cerebellar Cortex |
| 91 | 33.3 | 29 | 5 | | -36 | Right Middle Temporal Gyrus, Right Superior Temporal Gyrus |
| 68 | 36.1 | 13 | 33 | | -20 | Right Rectal Gyrus, Right Inferior Frontal Gyrus |
| 61 | 38.3 | -25 | -53 | | 32 | Brain Stem |
| ***Anxiety Main Effect*** | | | | | | |
| 88 | 15.2 | -3 | -99 | | 10 | Left Cuneus |
| 86 | 23.6 | 43 | 17 | | 18 | Right Insula |
| ***Trauma x Anxiety*** | | | | | | |
| 694 | 28.8 | 53 | 27 | | 10 | Right Middle Frontal Gyrus, Right Inferior Frontal Gyrus, Superior Frontal Gyrus |
| 122 | 19.7 | -43 | 47 | | 18 | Left Middle Frontal Gyrus, Left Superior Frontal Gyrus |
| 122 | 21.1 | -37 | -15 | | 62 | Precentral Gyrus |
| 90 | 29.1 | -29 | 35 | | 48 | Left Middle Frontal Gyrus |
| 79 | 15.6 | -27 | -53 | | -32 | Left Cerebellar Tonsil |
| 60 | 22.6 | 13 | -3 | | 44 | Right Cingulate Gyrus |
| 59 | 33.1 | 29 | -75 | | -28 | Right Uvula, Right Pyramis |
| 59 | 22.5 | -31 | -79 | | -12 | Left Fusiform Gyrus |
| ***Condition Main Effect*** | | | | | | |
| 3447 | 38.1 | 1 | -43 | | 48 | Bilateral Superior Parietal Lobule, Bilateral Precuneus, Right Middle Occipital Gyrus |
| 2178 | 37.3 | 7 | -31 | | 0 | Right Insula, Right Thalamus, Right Lentiform Nucleus |
| 487 | 30.2 | -29 | -77 | | -2 | Left Middle Temporal Gyrus, Left Inferior Occipital Gyrus |
| 339 | 21.7 | 1 | 61 | | 16 | Bilateral Medial Frontal Gyrus, Left Superior Frontal Gyrus |
| 302 | 27.2 | 43 | 33 | | 2 | Right Inferior Frontal Gyrus |
| 267 | 27.8 | -31 | 1 | | -8 | Left Lentiform Nucleus, Left Parahippocampal Gyrus |
| 179 | 30.9 | 41 | -55 | | 20 | Right Middle Temporal Gyrus, Right Superior Temporal Gyrus |
| 177 | 27.5 | -25 | 37 | | -10 | Left Inferior Frontal Gyrus, Left Middle Frontal Gyrus |
| 173 | 23.5 | 11 | -53 | | -6 | Right Declive, Right Culmen |
| 151 | 19.7 | -55 | -11 | | 36 | Left Precentral Gyrus |
| 146 | 20.9 | -1 | 1 | | 62 | Left Superior Frontal Gyrus. Right Medial Frontal Gyrus |
| 128 | 23.9 | 1 | -87 | | 30 | Bilateral Cuneus |
| 127 | 29.2 | 3 | -89 | | -4 | Bilateral Lingual Gyrus |
| 126 | 28.9 | -45 | 17 | | -4 | Left Inferior Frontal Gyrus, Left Precentral Gyrus |
| 118 | 23.0 | 7 | 39 | | -20 | Right Superior Frontal Gyrus, Right Medial Frontal Gyrus |
| 116 | 30.3 | 31 | -53 | | -22 | Right Culmen |
| 111 | 16.5 | -19 | 17 | | 48 | Left Middle Frontal Gyrus, Left Superior Frontal Gyrus |
| 100 | 25.8 | -29 | 5 | | 38 | Left Middle Frontal Gyrus |
| 98 | 30.2 | 57 | -41 | | 0 | Right Middle Temporal Gyrus |
| 95 | 26.4 | 47 | 7 | | -32 | Right Middle Temporal Gyrus, Right Superior Temporal Gyrus |
| 95 | 18.6 | 5 | -33 | | 26 | Right Cingulate Gyrus |
| 93 | 20.6 | -23 | 51 | | 2 | Left Superior Frontal Gyrus, Left Middle Frontal Gyrus |
| 91 | 19.5 | -9 | 41 | | -14 | Left Medial Frontal Gyrus |
| 82 | 27.3 | -21 | -61 | | -20 | Left Declive |
| 68 | 20.6 | 21 | -25 | | 24 | Right Caudate |
| 67 | 17.9 | 23 | -55 | | 66 | Right Superior Parietal Lobule |
| 65 | 19.9 | -17 | -19 | | -4 | Left Thalamus |
| 65 | 27.4 | -53 | 19 | | 24 | Left Inferior Frontal Gyrus, Left Middle Frontal Gyrus |
| 64 | 21.2 | 57 | -41 | | 22 | Right Superior Frontal Gyrus, Right Inferior Parietal Lobule |
| 64 | 19.5 | -1 | -25 | | 66 | Bilateral Medial Frontal Gyrus |
| 61 | 21.0 | 21 | 49 | | 4 | Right Medial Frontal Gyrus, Right Superior Frontal Gyrus |
| 61 | 17.1 | 21 | 17 | | 36 | Right Middle Frontal Gyrus, |
| 60 | 28.6 | 35 | -83 | | -6 | Right Inferior Occipital Gyrus, Right Middle Occipital Gyrus |
| 59 | 20.3 | -9 | 19 | | -6 | Left Anterior Cingulate |
| 59 | 21.8 | -35 | -47 | | 32 | Left Supramarginal Gyrus, Left Inferior Parietal Lobule |
| ***Trauma x Condition*** | | | | | | |
| 996 | 31.5 | -27 | -77 | | 46 | Left Precuneus, Left Superior Parietal Lobule |
| 766 | 36.5 | 49 | 15 | | 42 | Right Middle Frontal Gyrus |
| 593 | 40.0 | -3 | 27 | | -18 | Left Medial Frontal Gyrus, Left Superior Frontal Gyrus |
| 391 | 25.2 | 15 | 35 | | 0 | Right Medial Frontal Gyrus, Right Superior Frontal Gyrus |
| 210 | 32.3 | 33 | -77 | | 38 | Right Precuneus, Right Cuneus |
| 130 | 29.3 | -37 | -27 | | 22 | Left Inferior Parietal Lobule, Left Cingulate Gyrus |
| 130 | 22.3 | -33 | -45 | | 46 | Left Inferior Parietal Lobule |
| 111 | 54.8 | -11 | -91 | | -18 | Left Declive, Left Lingual Gyrus |
| 101 | 24.5 | 35 | -19 | | 0 | Right Putamen |
| 95 | 28.1 | 23 | 31 | | 52 | Right Superior Frontal Gyrus |
| 94 | 27.8 | -33 | -81 | | -14 | Left Fusiform Gyrus, Left Declive |
| 93 | 39.5 | 47 | -67 | | -34 | Right Pyramis, Right Cerebellar Tonsil |
| 91 | 30.0 | -45 | -59 | | 8 | Left Middle Temporal Gyrus |
| 84 | 23.1 | -25 | 3 | | -6 | Left Lentiform Nucleus, Left Parahippocampal Gyrus |
| 84 | 26.7 | -35 | 29 | | -8 | Left Inferior Frontal Gyrus |
| 80 | 25.8 | -3 | -47 | | 0 | Bilateral Cerebellum Cortex |
| 73 | 29.9 | 13 | -75 | | -24 | Right Declive, Right Pyramis |
| 73 | 21.7 | 65 | 5 | | 14 | Right Inferior Frontal Gyrus, Right Precentral Gyrus |
| 72 | 41.5 | -53 | 19 | | 24 | Left Inferior Frontal Gyrus, Left Middle Frontal Gyrus |
| 72 | 21.4 | -31 | 9 | | 28 | Left Middle Temporal Gyrus |
| 71 | 19.6 | -43 | -29 | | 14 | Left Superior Temporal Gyrus, Left Insula |
| 71 | 26.3 | -39 | -13 | | 32 | Left Precentral Gyrus |
| 62 | 27.8 | 9 | -101 | | 0 | Right Cuneus, Right Lingual Gyrus |
| 62 | 19.5 | -31 | 17 | | 36 | Left Middle Frontal Gyrus |
| 62 | 16.2 | -17 | -5 | | 42 | Left Medial Frontal Gyrus, Left Cingulate Gyrus |
| 59 | 25.4 | -9 | -25 | | 68 | Left Postcentral Gyrus, Left Precentral Gyrus |
| ***Anxiety x Condition*** | | | | | | |
| 138 | 19.0 | 61 | 9 | | 26 | Right Inferior Frontal Gyrus, Right Precentral Gyrus |
| ***Trauma x Anxiety x Condition*** | | | | | | |
| 234 | 33.2 | 51 | 1 | | -10 | Right Superior Temporal Gyrus, Right Middle Temporal Gyrus |
| 178 | 29.2 | -3 | -47 | | 0 | Left Posterior Cingulate |
| 105 | 15.1 | -11 | -101 | | 8 | Left Cuneus |
| 75 | 19.7 | 51 | 17 | | 36 | Right Middle Frontal Gyrus |
| 61 | 15.2 | 51 | 27 | | 26 | Right Middle Frontal Gyrus |
| **Performance Feedback** | | | | | | |
| ***Trauma Main Effect*** | | | | | | |
| 731 | 33.2 | 41 | -3 | | -2 | Right Insula, Right Lentiform Nucleus, Right Superior Temporal Gyrus |
| 392 | 38.5 | 27 | -17 | | -38 | Right Cerebellar Tonsil |
| 230 | 45.0 | -5 | 7 | | 30 | Bilateral Cingulate Gyrus |
| 167 | 28.0 | -3 | -61 | | -12 | Bilateral Declive |
| 153 | 40.5 | -59 | -29 | | -20 | Left Inferior Temporal Gyrus, Fusiform Gyrus |
| 152 | 20.5 | 31 | 55 | | -10 | Right Middle Frontal Gyrus, Right Superior Frontal Gyrus |
| 147 | 25.3 | 33 | 27 | | 24 | Right Middle Frontal Gyrus |
| 142 | 23.9 | 53 | -51 | | -4 | Right Middle Temporal Gyrus |
| 133 | 18.0 | 59 | 15 | | 20 | Right Inferior Frontal Gyrus, Right Middle Frontal Gyrus |
| 122 | 24.3 | 55 | 1 | | 16 | Right Precentral Gyrus, Right Inferior Frontal Gyrus |
| 119 | 24.3 | 35 | -25 | | 20 | Right Insula |
| 116 | 25.6 | 19 | 15 | | -2 | Right Caudate, Right Anterior Cingulate |
| 98 | 27.1 | 3 | -83 | | -6 | Bilateral Lingual Gyrus |
| 91 | 31.0 | 53 | -35 | | -24 | Right Inferior Temporal Gyrus, Right Fusiform Gyrus |
| 91 | 24.4 | 19 | 69 | | 10 | Right Superior Frontal Gyrus, Right Middle Frontal Gyrus |
| 87 | 18.3 | -9 | 69 | | 12 | Left Superior Frontal Gyrus |
| 84 | 19.8 | -33 | -69 | | -12 | Left Fusiform Gyrus, Left Inferior Occipital Gyrus |
| 83 | 20.1 | -9 | -57 | | -26 | Left Nodule, Left Cerebellar Tonsil |
| 80 | 21.7 | -7 | 29 | | 10 | Bilateral Anterior Cingulate |
| 79 | 45.5 | -13 | -11 | | -4 | Left Thalamus |
| 78 | 21.6 | 25 | -65 | | -30 | Right Cerebellar Tonsil, Right Pyramis |
| 77 | 27.9 | 53 | 5 | | 32 | Right Precentral Gyrus, Right Inferior Frontal Gyrus |
| 76 | 21.3 | -3 | 21 | | -22 | Bilateral Medial Frontal Gyrus, Bilateral Rectal Gyrus |
| 72 | 16.1 | 17 | -31 | | 36 | Right Cingulate Gyrus |
| 69 | 16.7 | 19 | -31 | | 58 | Right Postcentral Gyrus |
| ***Anxiety Main Effect*** | | | | | | |
| 99 | 23.2 | -21 | -49 | | 68 | Left Postcentral Gyrus |
| 81 | 20.3 | 57 | -35 | | 34 | Right Submarginal Gyrus, Right Inferior Parietal Lobule |
| 76 | 17.5 | 51 | 9 | | -4 | Right Superior Temporal Gyrus, Right Insula |
| 69 | 28.3 | -57 | 3 | | 12 | Left Precentral Gyrus |
| ***Trauma x Anxiety*** | | | | | | |
| 281 | 19.2 | 47 | 41 | | 22 | Right Middle Frontal Gyrus |
| 213 | 17.0 | 53 | 25 | | 24 | Right Inferior Frontal Gyrus, Right Middle Frontal Gyrus |
| 139 | 25.8 | -17 | -51 | | -28 | Left Cerebellum Cortex |
| 78 | 20.6 | 25 | -75 | | -12 | Right Lingula Gyrus, Right Declive |
| 64 | 40.0 | -41 | -11 | | 24 | Left Precentral Gyrus, Left Postcentral Gyrus |
| ***Condition Main Effect*** | | | | | | |
| 203 | 27.3 | -15 | -65 | | -28 | Left Pyramis, Left Declive |
| 129 | 14.7 | 31 | 33 | | 40 | Right Middle Frontal Gyrus |
| 113 | 18.2 | 41 | 41 | | 16 | Right Middle Frontal Gyrus |
| 106 | 17.1 | 47 | -1 | | 48 | Right Middle Frontal Gyrus, Right Precentral Gyrus |
| 69 | 278 | -45 | 3 | | -18 | Left Middle Temporal Gyrus, Left Superior Temporal Gyrus |
| ***Trauma x Condition*** | | | | | | |
| 3664 | 31.8 | 53 | 37 | | -4 | Bilateral Superior Frontal Gyrus, Bilateral Middle Frontal Gyrus |
| 570 | 40.3 | -13 | -63 | | -28 | Bilateral Pyramis, Bilateral Cerebellar Tonsil |
| 267 | 32.8 | 19 | -35 | | -36 | Right Cerebellar Tonsil |
| 234 | 25.0 | 17 | 15 | | 60 | Right Superior Frontal Gyrus, Right Middle Frontal Gyrus |
| 219 | 34.3 | -25 | 29 | | -22 | Left Superior Temporal Gyrus, Left Inferior Frontal Gyrus |
| 167 | 24.1 | 39 | 19 | | -24 | Right Superior Temporal Gyrus, Right Inferior Frontal Gyrus |
| 116 | 24.9 | 55 | -51 | | -2 | Right Middle Temporal Gyrus |
| 105 | 24.2 | -43 | -75 | | -10 | Left Middle Occipital Gyrus, Left Inferior Occipital Gyrus |
| 105 | 24.0 | -51 | -71 | | 8 | Left Middle Temporal Gyrus, Left Superior Temporal Gyrus |
| 83 | 20.1 | 43 | -65 | | -34 | Right Cerebellar Tonsil, Right Pyramis |
| 75 | 35.2 | -5 | -45 | | 6 | Left Culmen, Bilateral Posterior Cingulate |
| 74 | 19.5 | 57 | -5 | | 40 | Right Precentral Gyrus, Right Middle Frontal Gyrus |
| 73 | 43.7 | 69 | -1 | | 8 | Right Superior Temporal Gyrus |
| 63 | 24.7 | -31 | 27 | | 10 | Left Inferior Frontal Gyrus |
| 63 | 23.5 | 63 | -7 | | 26 | Right Precentral Gyrus |
| ***Anxiety x Condition*** | | | | | | |
| 201 | 29.9 | -51 | -27 | | 30 | Left Inferior Parietal Lobule, Left Postcentral Gyrus |
| 65 | 15.9 | -17 | -67 | | -30 | Left Pyramis, Left Cerebellar Tonsil |
| ***Trauma x Anxiety x Condition*** | | | | | | |
| 3177 | 30.8 | 53 | 11 | | -6 | Bilateral Middle Frontal Gyrus, Bilateral Superior Frontal Gyrus, Right Inferior Frontal Gyrus |
| 415 | 37.9 | -25 | 7 | | 2 | Left Lentiform Nucleus |
| 381 | 29.1 | 3 | -73 | | -26 | Left Pyramis, Left Cerebellar Tonsil |
| 196 | 18.7 | -5 | 23 | | 58 | Right Superior Frontal Gyrus, Left Superior Frontal Gyrus |
| 172 | 24.3 | -17 | 9 | | -30 | Left Inferior Frontal Gyrus |
| 130 | 19.5 | -19 | -101 | | 10 | Left Cuneus, Left Middle Occipital Gyrus |
| 123 | 19.2 | 19 | -73 | | -24 | Right Declive |
| 120 | 34.3 | -15 | 21 | | 12 | Left Caudate |
| 99 | 19.0 | 19 | -33 | | -36 | Right Cerebellar Tonsil |
| 83 | 20.9 | -45 | 19 | | -16 | Left Superior Temporal Gyrus |
| 70 | 21.2 | -31 | -97 | | 4 | Right Middle Occipital Gyrus |
| ***Performance Main Effect*** | | | | | | |
| 98 | 21.9 | -37 | -61 | | 26 | Left Middle Temporal Gyrus, Left Angular Gyrus |
| ***Trauma x Performance*** | | | | | | |
| 250 | 25.9 | 27 | -51 | | 44 | Right Superior Parietal Lobule, Right Inferior Parietal Lobule |
| 158 | 33.9 | -49 | -65 | | 12 | Left Middle Temporal Gyrus |
| 120 | 21.0 | -59 | -15 | | 6 | Left Superior Temporal Gyrus, Left Precentral Gyrus |
| 110 | 20.4 | 21 | -11 | | 68 | Right Precentral Gyrus, Right Superior Frontal Gyrus |
| 98 | 21.9 | 31 | -9 | | 46 | Right Precentral Gyrus, Right Middle Frontal Gyrus |
| 84 | 41.5 | -1 | -61 | | -16 | Bilateral Declive |
| 84 | 18.1 | 41 | -19 | | 46 | Right Postcentral Gyrus |
| 70 | 17.9 | -21 | -1 | | 48 | Left Middle Frontal Gyrus, Left Medial Frontal Gyrus |
| 65 | 17.0 | -39 | 21 | | -24 | Left Superior Temporal Gyrus |
| 61 | 188 | 7 | -67 | | -28 | Bilateral Uvula |
| ***Anxiety x Performance*** | | | | | | |
| 77 | 18.3 | 47 | -49 | | -4 | Right Middle Temporal Gyrus |
| ***Trauma x Anxiety x Performance*** | | | | | | |
| 398 | 38.8 | 47 | 9 | | 6 | Right Insula, Right Precentral Gyrus |
| 242 | 27.5 | -33 | 7 | | 12 | Left Insula, Left Precental Gyrus |
| 132 | 31.0 | -57 | -25 | | 44 | Left Inferior Parietal Lobule, Left Precentral Gyrus |
| 131 | 33.2 | -37 | 51 | | 30 | Left Middle Frontal Gyrus, Left Superior Frontal Gyrus |
| 62 | 21.1 | -49 | -59 | | 48 | Left Inferior Parietal Lobule, Left Superior Parietal Lobule |
| ***Condition x Performance*** | | | | | | |
| 107 | 24.1 | -29 | 31 | | 14 | Left Inferior Frontal Gyrus, Left Middle Frontal Gyrus |
| 71 | 18.4 | -31 | 15 | | 54 | Left Middle Frontal Gyrus, Left Superior Frontal Gyrus |
| 69 | 22.6 | -29 | 9 | | 0 | Left Lentiform Nucleus |
| ***Trauma x Condition x Performance*** | | | | | | |
| 705 | 44.8 | -25 | 29 | | -20 | Left Superior Temporal Gyrus, Left Middle Temporal Gyrus |
| 341 | 41.3 | -51 | 37 | | -6 | Left Inferior Frontal Gyrus |
| 272 | 36.7 | 3 | -69 | | -24 | Bilateral Pyramis, Bilateral Declive, Bilateral Uvula |
| 247 | 32.1 | -31 | 29 | | 22 | Left Inferior Frontal Gyrus |
| 245 | 31.4 | 55 | -49 | | -30 | Right Cerebellar Tonsil, Right Culmen |
| 153 | 18.5 | 45 | -25 | | 48 | Right Postcentral Gyrus |
| 122 | 22.7 | 47 | -69 | | -34 | Right Tuber, Right Cerebellar Tonsil, Right Pyramis |
| 120 | 25.7 | -29 | -53 | | 8 | Left Posterior Cingulate |
| 117 | 20.5 | 17 | -65 | | -4 | Right Lingual Gyrus, Right Culmen |
| 115 | 15.5 | -31 | 53 | | 18 | Left Superior Frontal Gyrus, Left Middle Frontal Gyrus |
| 100 | 22.3 | 31 | 3 | | -26 | Right Superior Temporal Gyrus |
| 96 | 24.4 | 5 | -49 | | 2 | Right Lingual Gyrus, Bilateral Culmen, Right Parahippocampal Gyrus |
| 89 | 16.2 | 49 | 37 | | -10 | Right Middle Frontal Gyrus, Right Inferior Frontal Gyrus |
| 79 | 25.7 | 23 | 19 | | 56 | Right Superior Frontal Gyrus, Right Middle Frontal Gyrus |
| 78 | 21.1 | -3 | -67 | | -16 | Bilateral Declive, Bilateral Culmen |
| 66 | 29.1 | -55 | -45 | | -30 | Left Tuber, Left Culmen |
| 65 | 18.4 | -53 | 25 | | 20 | Left Middle Frontal Gyrus, Left Inferior Frontal Gyrus |
| ***Anxiety x Condition x Performance*** | | | | | | |
| 189 | 19.3 | 13 | 31 | | 4 | Bilateral Anterior Cingulate |
| 169 | 27.0 | 29 | 37 | | 6 | Right Inferior Frontal Gyrus |
| 111 | 28.1 | -11 | 11 | | 16 | Left Caudate |
| 92 | 19.2 | -49 | -27 | | 32 | Left Postcentral Gyrus, Left Inferior Parietal Lobule |
| 79 | 21.3 | -29 | -35 | | 40 | Left Inferior Parietal Lobule |
| ***Trauma x Anxiety x Condition x Performance*** | | | | | | |
| 349 | 32.5 | -29 | 31 | | 14 | Left Caudate, Left Anterior Cingulate |
| 156 | 23.1 | -43 | 3 | | -24 | Left Superior Temporal Gyrus, Left Middle Temporal Gyrus |
| 100 | 26.4 | 39 | 7 | | -24 | Right Superior Temporal Gyrus |
| 98 | 19.3 | 5 | -79 | | -24 | Right Uvula, Right Declive |
| 93 | 31.1 | 23 | -49 | | -10 | Right Parahippocampal, Right Culmen |
| 91 | 26.6 | -25 | 39 | | 44 | Left Superior Frontal Gyrus |
| 90 | 21.0 | -29 | 21 | | 58 | Left Middle Frontal Gyrus, Left Superior Frontal Gyrus |
| 85 | 16.4 | -39 | 25 | | 4 | Left Inferior Frontal Gyrus |
| 72 | 18.8 | -39 | -11 | | -22 | Left Fusiform Gyrus, Left Inferior Temporal Gyrus |
| 65 | 19.6 | -37 | -51 | | 10 | Left Posterior Cingulate |
| **Right Ventral Striatum Seed** | | | | | | |
| **Reward Anticipation** | | | | | | |
| ***Trauma Main Effect*** | | | | | | |
| 145 | 26.4 | -29 | 63 | | 8 | Left Middle Frontal Gyrus, Left Superior Frontal Gyrus |
| 132 | 32.2 | 49 | -67 | | -34 | Right Pyramis, Right Tuber |
| 128 | 20.5 | -23 | 33 | | 2 | Left Anterior Cingulate |
| 126 | 32.6 | 23 | 67 | | 2 | Right Superior Frontal Gyrus, Right Middle Frontal Gyrus |
| 76 | 30.9 | -27 | -1 | | 50 | Left Middle Frontal Gyrus |
| ***Anxiety Main Effect*** | | | | | | |
| 105 | 25.2 | 51 | -67 | | -2 | Right Middle Occipital Gyrus, Right Fusiform Gyrus, Right Inferior Temporal Gyrus |
| 83 | 23.3 | 49 | -25 | | 50 | Right Postcentral Gyrus |
| 63 | 17.4 | 45 | -79 | | 0 | Right Middle Occipital Gyrus |
| ***Condition Main Effect*** | | | | | | |
| 97 | 26.0 | -37 | -71 | | 46 | Left Inferior Parietal Lobule, Left Superior Parietal Lobule |
| 87 | 26.6 | -5 | 51 | | -16 | Bilateral Medial Frontal Gyrus |
| 70 | 15.7 | 1 | 53 | | 18 | Bilateral Medial Frontal Gyrus |
| ***Trauma x Condition*** | | | | | | |
| 473 | 41.3 | -37 | -71 | | 44 | Left Precuneus, Left Inferior Parietal Lobule |
| 386 | 26.6 | 33 | 5 | | 34 | Right Middle Frontal Gyrus, Right Precentral Gyrus |
| 350 | 26.6 | 47 | -47 | | 46 | Right Inferior Parietal Lobule, Right Precuneus |
| 345 | 24.3 | -9 | 45 | | -4 | Bilateral Medial Frontal Gyrus |
| 170 | 23.0 | 17 | -61 | | 64 | Right Precuneus, Right Superior Parietal Lobule |
| 142 | 61.8 | 39 | -19 | | 18 | Right Insula |
| 133 | 20.2 | 39 | 15 | | 34 | Left Middle Frontal Gyrus |
| 114 | 23.9 | -31 | 57 | | -4 | Left Middle Frontal Gyrus, Left Superior Frontal Gyrus |
| 84 | 15.6 | -5 | 23 | | 42 | Left Superior Frontal Gyrus, Left Medial Frontal Gyrus |
| 82 | 22.6 | 33 | 7 | | 46 | Right Middle Frontal Gyrus |
| 77 | 41.6 | -55 | 25 | | 26 | Left Middle Frontal Gyrus, Left Inferior Frontal Gyrus |
| 76 | 22.1 | 9 | 3 | | 44 | Right Cingulate Gyrus, Right Superior Frontal Gyrus, Right Medial Frontal Gyrus |
| 74 | 16.5 | 61 | -51 | | 24 | Right Superior Temporal Gyrus, Right Middle Temporal Gyrus |
| 72 | 19.5 | -59 | -49 | | 20 | Left Superior Temporal Gyrus |
| 66 | 21.6 | -1 | 27 | | -16 | Bilateral Medial Frontal Gyrus, Bilateral Anterior Cingulate |
| 64 | 16.6 | -3 | 49 | | 34 | Bilateral Medial Frontal Gyrus |
| 64 | 19.4 | -25 | -19 | | 62 | Left Precentral Gyrus |
| 60 | 14.3 | -41 | -27 | | 12 | Left Superior Temporal Gyrus, Left Transverse Temporal Gyrus |
| **Performance Feedback** | | | | | | |
| ***Trauma Main Effect*** | | | | | | |
| 748 | 24.2 | 43 | -3 | | 26 | Right Middle Frontal Gyrus, Right Precentral Gyrus |
| 405 | 39.7 | -29 | -53 | | 28 | Left Inferior Parietal Lobule, Left Supramarginal Gyrus |
| 391 | 19.3 | -5 | 21 | | 48 | Bilateral Superior Medial Gyrus |
| 285 | 25.5 | -13 | 41 | | 16 | Left Middle Frontal Gyrus, Left Inferior Frontal Gyrus |
| 283 | 24.6 | 35 | 51 | | 2 | Right Middle Orbital Gyrus |
| 277 | 30.1 | -29 | 17 | | 34 | Left Middle Frontal Gyrus |
| 239 | 29.6 | -19 | -87 | | -10 | Left Inferior Occipital Gyrus, Left Fusiform Gyrus |
| 161 | 27.2 | -29 | 15 | | 36 | Left Precentral Gyrus, Left Middle Frontal Gyrus |
| 123 | 36.8 | 33 | 15 | | 0 | Right Insula |
| 119 | 20.4 | 47 | -49 | | 46 | Right Inferior Parietal Lobule |
| 87 | 15.6 | 9 | -1 | | 58 | Bilateral Supplementary Motor Area |
| 71 | 20.0 | -27 | 49 | | 22 | Left Middle Frontal Gyrus |
| 62 | 22.2 | -9 | 43 | | 32 | Left Superior Medial Gyrus, Left Superior Frontal Gyrus |
| 61 | 17.5 | -35 | -27 | | 56 | Left Precentral Gyrus |
| ***Trauma x Condition*** | | | | | | |
| 3401 | 53.1 | 19 | 55 | | 32 | Bilateral Middle Frontal Gyrus, Bilateral Superior Frontal Gyrus |
| 486 | 30.5 | -5 | 3 | | 64 | Left Middle Frontal Gyrus, Bilateral Supplementary Motor Area |
| 248 | 43.9 | -11 | -59 | | -26 | Bilateral Cerebellum |
| 136 | 24.6 | 1 | 71 | | -2 | Left Mid Orbital Gyrus |
| 98 | 27.2 | 67 | 1 | | 12 | Right Rolandic Operculum, Right Postcentral Gyrus |
| 94 | 24.8 | 9 | -77 | | -26 | Bilateral Cerebellum |
| 86 | 24.8 | -13 | -101 | | 6 | Left Superior Occipital Gyrus |
| ***Trauma x Anxiety x Condition*** | | | | | | |
| 782 | 30.3 | 19 | 49 | | 40 | Right Middle Frontal Gyrus, Right Superior Frontal Gyrus |
| 228 | 28.1 | -41 | 33 | | 44 | Left Middle Frontal Gyrus, Left Superior Frontal Gyrus |
| 161 | 26.0 | 55 | 41 | | 4 | Right Inferior Frontal Gyrus, Right Middle Orbital Gyrus |
| 102 | 23.9 | -23 | 59 | | 22 | Left Superior Frontal Gyrus, Left Middle Frontal Gyrus |
| 87 | 26.1 | -21 | -97 | | 14 | Left Superior Occipital Gyrus |
| 65 | 17.3 | -17 | 15 | | 62 | Left Superior Frontal Gyrus, Left Middle Frontal Gyrus |
| 60 | 22.5 | -47 | -59 | | -32 | Left Cerebellum |
| ***Performance Main Effect*** | | | | | | |
| 82 | 22.7 | 33 | 35 | | 40 | Right Middle Frontal Gyrus |
| ***Trauma x Performance*** | | | | | | |
| 337 | 28.7 | 45 | 31 | | 30 | Right Inferior Frontal Gyrus, Right Middle Frontal Gyrus |
| 298 | 28.4 | 33 | 15 | | 50 | Right Middle Frontal Gyrus |
| 188 | 31.6 | -27 | -95 | | 6 | Left Inferior Occipital Gyrus, Left Middle Occipital Gyrus |
| 153 | 25.6 | -43 | 51 | | 4 | Left Middle Orbital Gyrus |
| 104 | 30.3 | 19 | -93 | | -4 | Right Calcarine Gyrus, Right Inferior Occipital Gyrus, Right Middle Occipital Gyrus |
| 68 | 28.5 | 67 | -1 | | 14 | Right Postcentral Gyrus, Right Rolandic Operculum |
| 67 | 25.8 | 39 | 51 | | 2 | Right Middle Orbital Gyrus |
| ***Condition x Performance*** | | | | | | |
| 78 | 15.0 | 51 | 37 | | -12 | Right Inferior Frontal Gyrus |
| ***Trauma x Condition x Performance*** | | | | | | |
| 696 | 32.7 | 31 | 41 | | 38 | Bilateral Superior Frontal Gyrus, Bilateral Middle Frontal Gyrus |
| 212 | 20.1 | -45 | 39 | | -8 | Left Inferior Frontal Gyrus |
| 212 | 34.6 | -3 | 21 | | 62 | Left Superior Medial Gyrus, Left Superior Frontal Gyrus |
| 202 | 28.7 | 35 | -5 | | -42 | Right Inferior Temporal Gyrus |
| 185 | 38.1 | 65 | -5 | | 14 | Right Postcentral Gyrus, Right Rolandic Operculum |
| 160 | 23.9 | 25 | 13 | | 56 | Right Superior Frontal Gyrus, Right Middle Frontal Gyrus |
| 131 | 31.9 | 49 | -19 | | -22 | Right Inferior Temporal Gyrus, Right Fusiform Gyrus |
| 124 | 40.1 | 53 | -53 | | -16 | Right Inferior Temporal Gyrus |
| 120 | 46.1 | 47 | -59 | | -34 | Right Cerebellum |
| 113 | 39.9 | -31 | -59 | | -32 | Left Cerebellum |
| 105 | 32.5 | -55 | -57 | | -28 | Left Cerebellum |
| 101 | 24.9 | 49 | 39 | | -10 | Right Inferior Frontal Gyrus |
| 99 | 19.5 | 51 | -29 | | -28 | Right Cerebellum |
| 99 | 23.4 | -25 | 57 | | 22 | Left Middle Frontal Gyrus, Left Superior Frontal Gyrus |
| 97 | 21.2 | 59 | 3 | | -32 | Right Inferior Temporal Gyrus |
| 75 | 23.9 | -37 | -19 | | -4 | Left Hippocampus |
| 65 | 19.9 | 1 | 45 | | 28 | Bilateral Superior Medial Frontal Gyrus |
| ***Trauma x Anxiety x Condition x Performance*** | | | | | | |
| 78 | 18.6 | 35 | -5 | | -42 | Right Inferior Temporal Gyrus |
| 62 | 19.2 | 49 | -19 | | -22 | Right Inferior Temporal Gyrus |
